# NR5G-SAM: A SLAM Framework for Field Robot Applications Based on 5G New Radio

**DOI:** 10.3390/s23115354

**Published:** 2023-06-05

**Authors:** Panagiotis T. Karfakis, Micael S. Couceiro, David Portugal

**Affiliations:** 1 Ingeniarius Ltd., R. Nossa Sra. Conceição 146, 4445-147 Alfena, Portugal; micael@ingeniarius.pt; 2 Institute of Systems and Robotics, University of Coimbra, 3030-290 Coimbra, Portugal; davidbsp@isr.uc.pt

**Keywords:** 5G NR, SLAM, sensor fusion, pose estimation, field robotics, radio mapping, iSAM, REM

## Abstract

Robot localization is a crucial task in robotic systems and is a pre-requisite for navigation. In outdoor environments, Global Navigation Satellite Systems (GNSS) have aided towards this direction, alongside laser and visual sensing. Despite their application in the field, GNSS suffers from limited availability in dense urban and rural environments. Light Detection and Ranging (LiDAR), inertial and visual methods are also prone to drift and can be susceptible to outliers due to environmental changes and illumination conditions. In this work, we propose a cellular Simultaneous Localization and Mapping (SLAM) framework based on 5G New Radio (NR) signals and inertial measurements for mobile robot localization with several gNodeB stations. The method outputs the pose of the robot along with a radio signal map based on the Received Signal Strength Indicator (RSSI) measurements for correction purposes. We then perform benchmarking against LiDAR-Inertial Odometry Smoothing and Mapping (LIO-SAM), a state-of-the-art LiDAR SLAM method, comparing performance via a simulator ground truth reference. Two experimental setups are presented and discussed using the sub-6 GHz and mmWave frequency bands for communication, while the transmission is based on down-link (DL) signals. Our results show that 5G positioning can be utilized for radio SLAM, providing increased robustness in outdoor environments and demonstrating its potential to assist in robot localization, as an additional absolute source of information when LiDAR methods fail and GNSS data is unreliable.

## 1. Introduction

Simultaneous Localization and Mapping (SLAM) is a fundamental research topic in robotics [1,2], which significantly impacts prolonged operation of mobile robots. Several methods have been proposed to solve the SLAM problem, e.g., using Light Detection And Ranging (LiDAR) [3] and visual-based methods [4] to build a representative map, while maintaining the robot localized within that map. Traditionally, this is accomplished by obtaining the physical ranges between the robot and its surrounding objects or through the detection of suitable features to track within the target area.

Modern SLAM approaches utilize multi-modal sensory systems to perceive the robot’s environment [5]. In outdoor areas, uncertainty increases dramatically as mobile robots traverse uneven terrain and dense urban areas, such as densely structured cities but also rural unstructured environments, such as forests, mines and offshore. In these scenarios, visual and laser cues can be limited due to many factors: the homogeneity of the environment, juddering motion from rough terrain, illumination variations as well as temporary seasonal changes, e.g., fog, rain, wind, dust and smoke clouds.

Global Navigation Satellite Systems (GNSS)-denied outdoor scenarios are common due to the geometry of the environment, where multi-path and shadow fading affect the atmospherically received signal, especially in mountainous terrains and urban canyons. Moreover, urban structures are a source of obstruction for signals arriving from satellites by limiting the visible horizon of a receiver. Hence, the availability of fix measurements and their quality may be obsolete. The above factors introduce error into the localization system of GNSS-dependent robots and can be detrimental during outdoor navigation and mapping tasks.

To mitigate this problem, a terrestrial cellular Fifth Generation Mobile Network (5G) New Radio (NR) positioning method can provide significant advantages when coupled with standalone modalities, such as visual-, LiDAR-, inertial-, and radar-based approaches. It is also less prone to the risks identified for GNSS, as it benefits from 5G being ubiquitously deployed in urban and suburban areas around the world, with cell availability not being a major concern for positioning applications [6]. As such, it is foreseen to boost new industrial use cases for the domains of autonomous vehicles and robotics, such as healthcare, food production and agriculture, city management, public transportation, manufacturing, and asset maintenance.

In this study, we propose NR5G-SAM, a 5G NR SLAM framework which uses multilateration and Time of Arrival (ToA) ranging to generate a tightly coupled cellular radio-inertial pose estimation. This approach is grounded on the factor graph SLAM algorithm, described comprehensively in [7], which solves the full SLAM problem by estimating the robot’s localization, while mapping the environment and keeping track of the pose history as a set of graph nodes. Through an estimation of the Maximum A Posteriori (MAP) likelihood of the full SLAM problem, we create a sparse graph from the set of estimated robot poses, and perform iterative Smoothing and Mapping (SAM) [8,9] to improve the localization by a set of factors. The estimation of robot positioning is computed from the transmitted Positioning Reference Signal (PRS) between each gNodeB (gNB) base station to the User Equipment (UE), formally known as the Down-Link (DL) signals.

Channel State Information (CSI) can be complementary used with the goal to improve the estimation process and mitigate positioning errors [10]. Therefore, we create a radio map of the environment, consisting of Received Signal Strength Indicator (RSSI) measurements from each gNb, which can be utilized as features during navigation within the environment. The developed Radio Environmental Map (REM) is used to determine the relative location of the robot to the gNB stations, by comparing the ideal signal propagation model with the estimation at the UE side, similar to the Inverse Distance Weighted (IDW) interpolation technique. The proposed mapping technique finds inspiration in fingerprinting approaches [11,12], with the difference being that it is performed online using the 5G Urban Macro-Cell (UMa) models’ equations [13] and with no prior collection points stored in a database. The radio maps serve as a simple representation of the signal propagation within the environment.

We validate the 5G SLAM approach under two experimental setups, using the FR1 n78 band and the FR2 n257 band frequencies over a large perimetric trajectory within a simulated agricultural environment, using the UMa propagation model [14]. In order to demonstrate its significance, we compare NR5G-SAM with LIO-SAM [15], a state-of-the-art LiDAR-inertial SLAM framework to highlight the advantages and disadvantages it can potentially provide to the mobile robotics domain. This work is an extension of our previous work on 5G Positioning [16] and serves as an important development step towards an innovative and robust cellular SLAM framework for field robotics.

The contributions of this work aim to answer fundamental questions of our ongoing research on the potential of using 5G NR for field robot localization. Namely:

- Which are the relevant attempts for radio localization and mapping in field robotics?
- Can we perform localization assisted by cellular 5G NR signals and Millimeter Wave (mmWave) and what accuracy can we expect?
- Can we use 5G NR CSI to interpolate REMs at a given geographic location?
- Can we use 5G NR coupled with other sensor modalities for localization and a REM as a radio SLAM framework?
- How would the NR5G-SAM framework perform compared to a state-of-the-art LiDAR SLAM approach in a relevant outdoor environment?

Our aim is to highlight that solely LiDAR-Inertial approaches are not sustainable on their own for long term localization in outdoor environments. The focus of this work is not to demonstrate the superiority of NR5G-SAM over LIO-SAM, but rather showcase the limitations of a LiDAR-Inertial approach in challenging outdoor environments, which is a commonly deployed sensor setup in autonomous vehicles and field robots globally and specifically cover the scenarios that GNSS is impaired or denied. Although the performance comparison may not seem entirely appropriate, we firmly believe in its relevance within the context of the paper’s research objectives, especially for real-world robot applications.

In the remainder of this document, we describe related work on radio technologies used for localization, mapping, and SLAM in Section 2. We also provide as a contribution a general survey of related works in this area based on relevant radio technologies. In the following Section 3, we present the design and implementation of the NR5G-SAM framework and in Section 4 we describe in detail the 5G FR1 and FR2 experimental test-beds. In Section 5, we present our experimental results and critically analyze the outcome of the 5G positioning and the proposed NR5G-SAM algorithm compared to the LIO-SAM approach. As a projection of our current outcomes, we critically analyze the challenges that lie in the future steps of this research in Section 6. Finally, in Section 7, we draw our conclusions and present the future research direction of 5G NR SLAM within field robotics.

## 2. Related Work

In this section, we introduce relevant radio technologies that have been used for robot localization including 5G NR, and then emphasize on radio SLAM approaches and highlight their significance for field robotic applications.

### 2.1. Radio Technologies and 5G NR

Several radio-based technologies have been used for positioning purposes, including Bluetooth Low Energy (BLE), Ultra Wideband (UWB), Wireless Fidelity (WiFi), Long-Range Protocol (LoRa), and 4G Long-Term Evolution (LTE) [17,18,19,20,21] with varying degrees of success. Currently, 5G is a study item for the Third Generation Partnership Project (3GPP) Release 17, with the potential to not only outperform GNSS, but also enable accurate estimations of orientation [22]. 5G NR provides higher speeds, lower latencies, and larger bandwidth capacities than 4G-LTE [23], which can be very attractive for field robotic applications and the future of the Internet of Things (IoT), as Mobile Edge Computing (MEC) is now a reality in many regions around the world.

*Radio-based localization* occurs in two steps. Firstly, the radio waves need to be converted to ranges and/or angle information, e.g., using range-free or range-based approaches [24]. Secondly, depending on the environment topology and application requirements, triangulation, trilateration, multilateration, proximity, and scene analysis can be adopted to obtain the pose information for tracking purposes. An in-depth analysis of the methods can be seen in [25,26].

GNSS is commonly used for positioning with outdoor robots. However, the signal quality is not always available, especially in rural areas such as dense forests and canyons, but also in building-congested cities. With the high degree of resolvability of propagation paths in mmWave frequencies, multi-path information can be exploited in 5G both for positioning and mapping of the environment. Ray tracing is a popular technique that has given promising results for mmWave signals at the cost of additional computational resources [27]. The general requirements to achieve high accuracy radio localization in 5G cellular are: (i) very large data-to-transmission rates; (ii) application-dependent low latency; (iii) seamless Physical Layer (PHY) integration; (iv) low computational overhead; (v) continuous tracking without loss of frames; and (vi) recovery mechanisms.

Several 5G-based localization challenges have been identified in the past [28] including: (i) signal reflection, (ii) radio interference, (iii) obstruction, (iv) multi-path, (v) limited coverage, and (vi) cell resource loads at peak user times. In [29], the authors exploited the mmWave band of 5G to provide precise position information for location-based services considering the deployment costs and ease of installation. The algorithm was based on Bayesian tracking filter, which makes use of Multi-Path Components (MPC) to provide position information. Emphasis was given to the data association as a core block, which was used to associate the arrival time of each MPC to the predicted delay. A Maximum Likelihood Estimator (MLE) was used to estimate the arrival time from the received 5G signal. Moreover, the predicted delay was then computed from the positions of a Virtual Anchor (VA), which is the occurrence point of a signal reflection, and the estimated user position. The output of the data association block was given as an input to the Bayesian filter to continuously track the user position with a resulting Line-of-Sight (LoS) error of 2–3 cm in indoor setups.

In [30], joint positioning and clock synchronization in 5G Ultra-Dense Network (UDN) using a two-stage Extended Kalman Filter (EKF) was proposed. The model considered a high-density Base Station (BS) with Multiple-Input Multiple-Output (MIMO) arrays. This allows the UE to have a LoS with multiple BS for most of the time, thereby increasing the accuracy of the cascaded EKF system. The first stage of EKF active on each BS, tracks the Direction of Arrival (DoA) and ToA of incoming uplink signals from UE. The second stage that runs on a central node in the cloud, estimates the position of the UE by fusing the DoA and ToA estimates from multiple BS. In addition, the clock drift between UE and network can be obtained. Results show that their model can achieve a sub-meter level positioning accuracy and a nano-second level network synchronization using a dedicated edge localization server.

Higher propagation losses at mmWave frequencies can degrade the localization accuracy in 5G NR networks. Abu-Shaban et al. [31] derived the Cramér–Rao Lower Bound (CRLB) for position and orientation errors. The model consists of a single BS with multiple antennae at a height of 10 m and a UE with multiple antennae operating at a carrier frequency of 38 GHz, rotated by 10 degree interval step. The Fisher Information Matrix (FIM) is computed for the Angle of Arrival (AoA), Angle of Departure (AoD), and ToA of multi-path signals, from which the error bounds for position and orientation are then computed. Results show that the Up-Link (UL) and DL signals are not identical. UL positioning is sensitive to UE orientation, whereas DL is not. In UL, the UE produces narrow beams travelling towards the BS. However, these beams might not even reach the BS because of the UE orientation at any given time, thereby decreasing the localization accuracy. They also showed that the mmWave systems can estimate the state of UE with a sub-meter position error and sub-degree orientation error for a BS-UE separation distances of up to 50 m.

A model to investigate the localization accuracy achieved by 5G NR is described in [32] for a Connected Autonomous Vehicle (CAV) use case, in which the CAV is not assumed to be time synchronized with the BS. A Two-Way Localization (TWL) method is proposed for this scenario based on two protocols. In the first protocol, Round-Trip Localization Protocol (RLP), the BS sends a signal to the CAV, which in turn responds with another signal after a pre-agreed time interval. Positioning is performed by considering only the DL signal. In the second protocol, Collaborative Localization Protocol (CLP), the CAV sends back the response to the BS and localization is achieved with both the transmitted and received signals by the BS. Thereafter, the Position Error Bound (PEB) and Orientation Error Bound (OEB) for both protocols are determined. Numerical results show that the CLP outperforms RLP in terms of error bounds. Additionally, the authors prove that having more antennae on the BS is more beneficial than having more antennae on the UE.

In [33,34,35], a model to estimate the speed, direction, and position of a moving UE based on a time reversal technique of focused beams and the effect of the transmitted power is proposed. Based on the focusing beam of a 5G MIMO system and the received signal by the UE, the Auto Correlation Function (ACF) is computed. Thereafter, the localization algorithm computes the UE speed, direction, and position by taking the ACF of the received signal and considering the geometrical positions of BS as inputs. A positioning accuracy of 0.2 m and orientation accuracy of 1.8${}^{\circ }$ is achieved through simulations.

In 5G networks, as with general radio-based mapping approaches, it is possible to create a *map of the environment* in order to assess the signal propagation and coverage of the cellular network. A radio map represents the signal quality between the BS and how a receiver perceives the network capabilities. An appropriate representation of radio maps, that has been a topic of research over the years in the fields of geodesy and telecommunications, are REMs [36], which can provide insights on how the radio signals travel in a given geographical area and how the obstacles limit coverage. REMs are built using either direct approaches, e.g., via interpolation methods [37,38,39,40] or indirect approaches without requiring the knowledge of the actual transmitter locations, e.g., LiVE [41], Signal to Noise Ratio (SNR)-aided methods [42], and Hybrid schemes [43].

Building a radio map with spatial coherence is an important and resource-intensive task, similarly to other robotic mapping processes based on visual and LiDAR features, such as varying image intensities or edge and plane information from laser ranging. A representation of the Received Signal Strength (RSS) of a radio transmission can be obtained with a robot carrying a receiver, while assessing a set of communication parameters, also known as CSI, which provide signal quality and power metrics during the reception and transmission of radio signals [11].

For the mmWave, the authors of [44] use 5G signals at 28 GHz with a bandwidth of 400 MHz to attain high-accuracy ranging resolution, performing mapping with a ray tracing approach [27]. The UE acts as a joint communication and radar system that scans the indoor environment while performing beam-forming [45] across different directions and observing the reflections from the environment.

A static grid-based approach and an advanced dynamic tracking approach were tested. The resulting map consists of the reflection points on walls and other objects during navigation inside a corridor and draws similarities to LiDAR mapping approaches, with high-frequency radio waves instead of occupancy.

Similarly, Azpilicueta et al. [46] developed a mapping framework in an environment consisting of indoor and outdoor transitions, where the 5G mmWave signal propagation and channel characteristics of the 28, 30, and 60 GHz frequencies were studied. The propagation mapping was analyzed in terms of the distribution appropriateness and based on a deterministic multi-module three-dimensional ray tracing code. They concluded that the (i) beam width, (ii) frequency band allocation, (iii) Modulation Carrier Scheme (MCS) election, (iv) antenna orientation and (v) node location affect the 5G mmWave signal. Furthermore, the alteration of the interference distributions can occur within a given application scenario not only during transitions from indoor to outdoor. In indoor and outdoor environments, it is shown that signal blockages from walls and shadowing effects can also be introduced by the human body.

It is foreseen that 5G systems will bring attractive features for *robot localization*, as they can provide centimeter accuracy, which is considerably more attractive than the standard state of sensing based on GNSS data or even other available radio-based solutions. In localization applications with 5G NR signals, applicability, latency, reliability, accuracy, and cost need to be taken into account. Autonomous vehicles are a clear use case that started to benefit already from 5G networks for platooning and safety, which share many similarities with mobile robots [47,48]. Another prominent use case are Unmanned Aerial Vehicles (UAVs), which have attracted attention for surveillance and asset monitoring tasks, as well as IoT applications [49].

### 2.2. SLAM

Most SLAM approaches based on wireless technology have been used for indoor applications, with the notable exception of outdoor GNSS-based SLAM [50]. Below, we review important body of research on *radio-based SLAM*.

Among works that use UWB technology, one of the most notable is [51], which employs a Factor SLAM approach that considers the association of spectral MPCs and their geometric features. A Back Propagation (BP)-SLAM algorithm was used for efficient marginalization of the joint posterior distribution between the real anchors and virtual anchor positions. A particle filter was utilized for the state estimation of an agent in an indoor area, with the reported Root Mean Square Error (RMSE) below 0.2 m. Similarly, the Channel-SLAM approach proposed in [52] simultaneously estimated the receiver position and the positions of the virtual transmitters with a Rao-Blackwellized Particle Filter (RBPF) using UWB. The idea behind this work was the assumption that MPCs are LoS signals, originating from the so-called VAs. The validation was performed against the posterior Crammer-Rao lower bound in an indoor scenario. In [53], the authors used a static UWB anchor and a mobile pedestrian carrying a hand-held UWB tag and an Inertial Measurement Unit (IMU). A positioning accuracy near 1 m was achieved 95% of the time during experiments. The MPC reflection points allow for the identification of objects and walls, comparing it with an existing floor plan map.

In [18], the UWB tag provides range measurements between the robot and anchors, which are used to update the Particle Filter (PF) weights. The proposed algorithm also includes a data association step to match the incoming range measurements with the corresponding anchors. The particle resampling was performed based on the importance sampling technique and the number of particles was adaptively adjusted to maintain the required accuracy level. The experimental results showed that only the proposed range (RO-SLAM) algorithm outperforms the traditional approaches in terms of accuracy and efficiency, at the cost of the computational overhead. The RadarSLAM algorithm was proposed in [54]. It utilizes a UWB super high-frequency pulse-echo radar sensor and a mobile robot equipped with a laser rangefinder for benchmarking purposes. The approach successfully converts the signals generated by the pulse-echo radar measurements to spectrograms, subsequently producing unique fingerprints of locations in the indoor environment. Moreover, Kadambi et al. [55] proposed an unsupervised neural Radio Frequency (RF) SLAM method from unlabeled CSI samples in a MIMO setup using Reconfigurable Intelligent Surfaces (RIS), implementing a Multi-Layer Perceptron (MLP) with Time of Flight (ToF) signals as input. The 3D map representation is visualized as a pointcloud, where each point is color-coded based on its CSI magnitude. In [56], the exchange of radio signals between the mobile agents and static anchors using MPCs was studied. A multiple VA-based RF-SLAM method was used to jointly estimate the time-varying location of agents and the static locations of flat surfaces. The reported theoretical accuracy was 0.1 m on average in the estimation of the agents and the VAs alike.

The work of Ismail et al. [57] demonstrated how existing WiFi infrastructure can be exploited for localization and mapping with low-power and low-computation hardware. In this experiment, a mobile robot equipped with a 3D LiDAR, a set of smartphones, and wheel encoders was used to perform similarity and loop closure through a two-step graph optimization process, using the WiFi signal fingerprints and allowing the mobile robot to navigate and correct its odometry drift. This method was able to attain a mean localization error of 0.88 m in a large industrial campus. A bearing-based WiFi SLAM approach for indoor robots was proposed in [58]. WiFi is integrated as a sensor to simultaneously locate the robot and estimate the WiFi Access Point (AP) positions in the environment using the complete CSI information and providing an inexpensive solution for indoor navigation using WiFi sensors. On the other hand, a Growing Gaussian Mixture Regression (GGMR) algorithm to simultaneously build a spatial map and a radio map was described in [59], which used a communication-based SLAM framework for indoor environments using mmWave signals. The method was tested in a simulation and the outputs were a signal power map and the position relative to the AP.

In [60], a rescue robot mapped the WiFi signal strength in the working area, correcting the drift of its six Degrees of Freedom (DoF) gyro-odometry localization system. Gaussian-based WiFi localization was extended to full three-dimensional and the proposed approach comprised a three-stage SLAM process that involved: (*i*) mapping of RSSI values within the environment; (*ii*) Gaussian process learning; and (*iii*) WiFi localization. Additional sensors were also contemplated, such as an IMU for attitude information and a 2D rangefinder. An improved WiFi GraphSLAM algorithm based on signal strength was implemented in [19] and was suitable for a broader range of environments since it made no signature uniqueness assumptions and uses solvers that are more tractable to larger map sizes.

**Figure 1:**
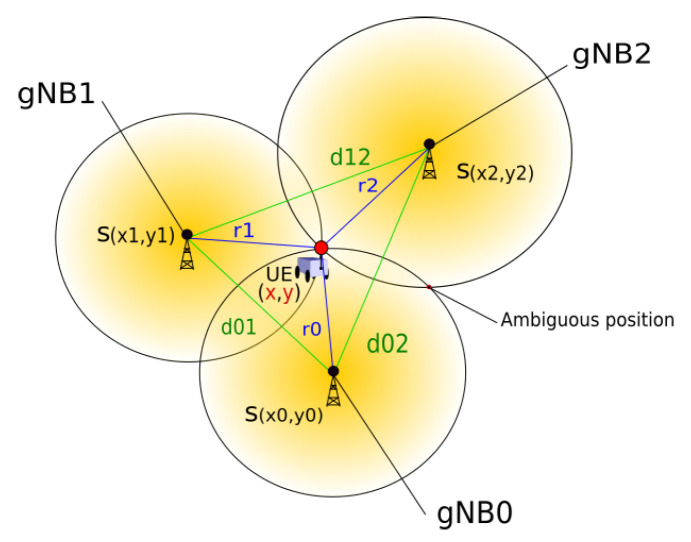
Positioning with 5G NR and User Equipment (UE) by performing trilateration between gNBs and the UE on a mobile robotic system [16].

Furthermore, 5G NR technology can be targeted to solve the SLAM problem, especially under GNSS-denied environments. However, only a small number of *5G SLAM* works have been proposed so far. Given a set of gNB stations placed over large kilometric distances, position tracking can be seen as a non-linear combinatorial problem [16]. A robot carrying UE may use 5G NR to track its position over time by comparing the ranges from each base station and infer its position, as shown in Figure 1. The ranges to each gNB $S_{n}\left(x,y\right)$ where *n* is the individual index can be either estimated from the UL and DL Time Difference of Arrival (TDoA) or ToA signals and quality information. In this context, inertial navigation systems can complement the system with the necessary angular information to estimate the orientation of the robot [19,57] and create a pose estimate through sensor fusion. Also, fingerprinting methods allow one to compare the current observation with the fingerprint history or database to identify areas with similar signal qualities [61].

A 5G mmWave SLAM approach was presented in [62]. Context inference was used to detect obstacles and their type in the simulated indoor environment, based on the reflected signals, which act as mirrors of the transmitted and the authors concluded that by increasing the number of APs generally improves the localization and obstacle detection functions, assuming enough RSS, AoA, and ToA measurements. Kim et al. [48] highlighted that with 5G mmWave signals it is possible to estimate the state of a vehicle (position and heading), from the transmissions of a single base station. They proposed a Bayesian 5G mmWave tracking filter, which explicitly relies on mapping the radio environment. The filter also acknowledged clock bias. Furthermore, a low-complexity multidimensional Estimation of Signal Parameters by Rotational Invariance Technique (ESPRIT)-based channel estimator applied to a 5G SLAM framework [63] and further described in [64] can provide the orientation of the UE in relation to the gNB stations.

The potential to jointly estimate the positions of UE and map their propagation environment using a single base station was explored in [65]. The algorithm was designed to work in a diffuse multi-path channel environment where the orientation and the clock bias were unknown. By utilizing only the diffuse multi-path information, the algorithm allowed for robust operation. In addition, the statistical dependency between CSI and the position, orientation, and clock offset of a UE in a 5G network was studied in [66], allowing one to infer the state of a UE and map its propagation environment. According to [67], 5G systems operating above 24 GHz have promising properties for localization and mapping. They proposed tackling the SLAM problem in four phases: (*i*) DL data transmission; (*ii*) multi-dimensional channel estimation; (*iii*) channel parameter clustering; and (*iv*) SLAM based on a novel likelihood function. The proposed approach was able to decompose the problem into simpler steps, thus leading to lower overall complexity. In the simulation, their ESPRIT variants utilized the same signal subspace to extract angular frequencies in all dimensions, allowing for the usage of the same channel distributions in the likelihood function, hence producing a new faster channel estimator for 5G SLAM with a low computational complexity.

In recent years, there has been significant progress in the development of efficient, consistent, and robust visual- and LiDAR-based SLAM algorithms, which have emerged as the leading approaches [15,68,69,70,71,72,73]. These methods utilize a combination of camera and LiDAR sensors to create high-quality maps of environments combined with accurate pose estimators. With the use of deep-learning techniques for feature extraction and localization, significantly improved the accuracy and robustness of these algorithms. Furthermore, recent advances in optimization methods, such as nonlinear pose graph optimization, enhanced the consistency and efficiency of the algorithms. Overall, these developments led to the increased adoption of visual- [4] and LiDAR-based [3] SLAM algorithms in various real-world applications within the robotics and autonomous vehicle communities. Most importantly, scene recognition capability is an important aspect of any algorithm, as accurate location information related to detected landmarks can be leveraged as absolute references. Yet, in radio SLAM methods it is harder to recognize revisited places through the feature extraction of features as signal propagation is spatiotemporarily dynamic.

Substantial progress in the development of efficient, consistent, and robust *visual- and LiDAR-based SLAM* algorithms has been witnessed in the latest decades [15,68,69,70,71,72,73]. Scene recognition capability is an important aspect of any SLAM algorithm, as accurate location information related to detected landmarks can be leveraged as absolute robot locations. Yet, in radio localization methods it is harder to recognize revisited places through feature extraction of landmarks as in visual SLAM approaches [4], and future work is foreseen towards this direction.

In this work, we address the full SLAM problem in which we are interested in generating a map, while estimating the posterior probability of the robot’s full trajectory *x* and map *m* given the sensor measurements *z* and control inputs *u* over time, $p(x_{1:t},m|z_{1:t},u_{1:t})$, as opposed to online SLAM, which considers only the most recent pose for state estimation. While conventional SLAM builds a geometrical map of the environment (e.g., see [74]), we use cellular RSSI measurements to obtain a mapping between signal strength measurements [11,75] and the locations of the base stations that transmit them. We represent the signal propagation via REMs, which are useful as part of the research around radio-based SLAM approaches [76,77], having the potential to assist robot localization [78].

## 3. Methodology

In this section, we describe the design and development of the proposed NR5G-SAM framework, including the main concepts and modules which form the proposed SLAM architecture.

### 3.1. NR5G-SAM System

The system receives sensor data from the 5G NR network, which we consider to be a 5G Stand Alone (SA) deployment and time synchronized, as well as the simulated onboard IMU. Additionally, it integrates wheel odometry velocity measurements. By using the measurements as observations, it keeps track of the robot within the environment and estimates its trajectory over time. Robot tracking is a non-linear optimization problem that can be formulated as a least squares process of computing the posterior state and is analogous to MAP. Factor graphs provide benefits by modeling the problem in a decoupled way, which allows for a modular design for SLAM algorithms, with a reference in several works such as [7,15]. Hence, we adopt it to model the 5G NR signal and sensor measurements as factors.

Three factors have been introduced within the framework, namely: (*i*) NR 5G factors; (*ii*) IMU pre-integration factors; and (*iii*) RSSI factors. All of these factors are binded by the robot state vector. We add a new node to the graph for every measurement that is received over time and we assume that all noise from the measurements and motion of the robot is Gaussian noise. In general, it is a good fit for many types of natural and human-made noise sources, such as electronic and thermal noise in sensors and also for atmospheric disturbances. Hence, we have assumed that the noise present in sensors and the 5G signals follows the Gaussian distribution as a practical approximation of their complex noise models. Additionally, it can account for the effects of multiple sources of noise that combine to produce the total noise effect on the robot’s state estimation process, present in many real-world phenomena and promotes the use of statistical estimation methods for modeling such as factor graphs. The generated factor graph is used to predict the localization of the robot through incremental smoothing and mapping (iSAM2) [9].

**Figure 2:**
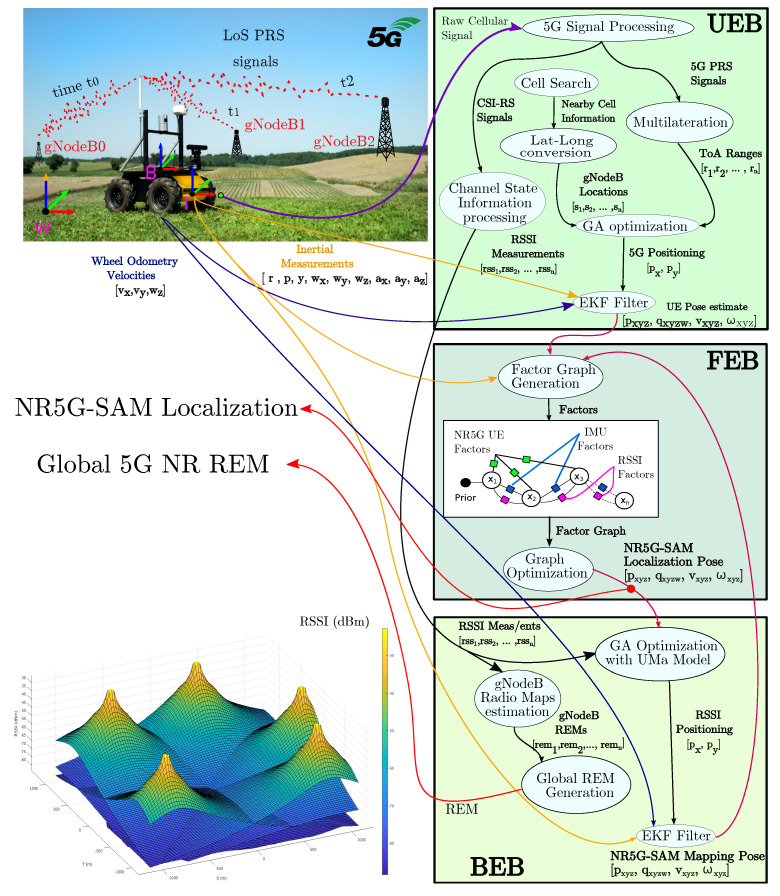
System architecture of NR5G-SAM consisting of three core components: (i) UEB, which deals with the UE signal reception and positioning; (ii) FEB, which performs factor graph optimization and computes the localization pose; and (iii) BEB, which addresses radio mapping, based on RSSI measurements of each gNodeB station, then generates the global REM and mapping pose estimation back into the FEB.

The overall SLAM framework consists of three core blocks. The User Equipment Block (UEB) concerns the signal reception and pre-processing of the CSI signals and DL signals and converts them into positioning information. The Front-End Block (FEB) is responsible for generating the factor graph and the robot localization by considering IMU and either NR5G or RSSI factors. The Back-End Block (BEB) is responsible for computing the individual radio environmental maps, via interpolation from RSSI measurements and the 3GPP UMa propagation model during navigation. This process generates a mapping pose estimate that is integrated within the FEB block for state prediction and corrections. As a representation of the fading signal, we also output a global 5G NR REM from the estimated individual radio maps. The full architecture is illustrated in Figure 2 and the analysis follows for each block in the forthcoming subsections.

### 3.2. User Equipment Block (UEB)

The PRS and CSI signals transmitted by the gNB stations are received by the UE on the robot via the DL communication channel and generated post-signal processing as follows: the received signals pass through an Analog-to-Digital (A/D) converter and then through Fast Fourier Transform (FFT) conversion to the frequency domain. The outcomes of this processing step are Orthogonal Frequency-Division Multiplexing (OFDM) signals containing the data. In our case, the CSI-RSSI and PRS signals are the information required and decoded using the cyclic prefix.

In this study, we consider that LoS signals exist between gNbs and the robot within the agricultural area. Range estimation is based on ToA, and the gNBs are assumed to be synchronized via a global network time reference. The ToA ranges are then used for multilateration to estimate the ranges $\left[r_{1},r_{2},\ldots ,r_{n}\right]$ from each base station to the robot, which includes additive noise that is assumed to follow a Gaussian distribution. The Channel State Information Reference Signal (CSI-RS) information is extracted from the CSI signals and vectorized as a set of RSSI $\left[rssi_{1},rssi_{2},\ldots ,rssi_{n}\right]$ values from each gNB station, measured in Decibel Milliwatts (dBm) and available as communication parameters in cellular communications, including 5G NR.

The locations of the stations in Cartesian space $\left[s_{1},s_{2},\ldots ,s_{n}\right]$ are obtained from a cell search operation that detects and receives basic location information regarding the gNBs in the surrounding area. An optimization of the ToA ranges is performed using a Genetic Algorithm (GA) to minimize the horizontal error during multilateration. Our previous developments [16] involved benchmarking three meta-heuristics for 5G positioning. The GA implementation provided superior results in terms of the mean execution time and average Euclidean error, thus becoming a natural design choice.

Station locations and the estimated ranges are used as inputs for the GA, which aims to find the optimal solution within the search space of potential 2D robot positions by considering the locations of the fixed gNB stations. This optimization step is required to account for time differences in signal reception and also for the multilateration errors in ranges.

The output of the GA’s fitness function for each estimated position is used as an uncertainty measurement, and the geometric evaluation of the ranges and analysis of the error correlation is performed using inverse trigonometric functions with the position and the gNBs. Practically, the 3D position estimation improves the GA’s performance compared to the 2D GA estimation, so the x- and y-coordinates are obtained from the estimated 3D vector, while the vertical z error is excluded until further mitigation methods are considered (see Section 4). The correlation between the positioning error in x and y and the fitness function’s output along each axis, are used for the meaningful estimation of a dynamic covariance matrix required by the EKF.

An experimental scaling factor is applied to convert the positioning fitness into covariance. This allows the EKF to consider the uncertainty of the 5G positioning estimation and fuse it with onboard IMU and robot velocity measurements. The output of the sensor fusion process includes the full pose of the robot, consisting of 5G-based position $\left[p_{x},p_{y},p_{z}\right]$, IMU orientation in quaternions $\left[q_{x},q_{y},q_{z},q_{w}\right]$, and wheel odometry linear and angular velocities $\left[v_{x},v_{y},v_{z},\omega_{x},\omega_{y},\omega_{z}\right]$. The EKF [79] generates the UE pose by smoothing the noisy 5G positioning signal and serves as an absolute measurement in the world frame *W* for the FEB.

### 3.3. Front-End Block (FEB)

Factor graph optimization is a method for estimating the MAP state estimate of a system by modeling it as a graph composed of nodes and edges. A factor is a function that defines the relationship between graph variables. Nodes represent the variables (such as robot poses and landmark positions) and edges represent the constraints (such as the measurements between landmarks and robot poses or between different landmarks). The factor graph is used to update the beliefs about the variables based on new measurements and to estimate the most likely values for all variables.

The inputs of the FEB are the UE pose estimates and IMU data. The factor graph models the relationships between the estimated position, velocity, and orientation of the system based on the pre-integrated IMU measurements and the nodes from the UE pose data.

The process of factor graph optimization involves iteratively minimizing the objective function that measures the discrepancy between the predicted and measured state vector of the system. This is typically conducted using a numerical optimization algorithm such as Levenberg–Marquardt [80] or Gauss–Newton. In our case, we perform it via smoothing and mapping (iSAM2), where the aim is to obtain the posterior pose distribution of the robot $X^{*}$ given the noisy sensor measurements and further model them through the iterative factorization of a set of factors ϕ(X), as per Equation (1). The objective function is described by Equation (2) and performs the MAP inference step of the factorization:

(1) $$P\left(X|Z\right)\propto \prod_{i}\phi_{i}\left(X_{i}\right).$$

(2) $$X^{*}=\underset{X}{\arg\max}\prod_{i}\phi_{i}\left(X_{i}\right).$$

Factor graph optimization typically includes a state transition model that describes how the state of the system evolves over time and a measurement model that is based on the sensor updates, provided by the IMU, UE, and RSSI pose data. The objective function often includes terms that penalize deviations from the observed states and encourage smooth trajectories of the estimated state variables. Equation (2) can be formulated as the equivalent Least Squares (LS) problem as represented in Equation (3), where $l(X_{i})$ is the sensor model likelihood, *z* is the measurement, and ∑ is the covariance, which are associated with $X_{i}$. By minimizing this equation, we iteratively obtain the updated state estimates:

(3) $$X^{*}=\underset{X}{\arg\min}\sum_{i}{∥l\left(X_{i}\right)-z_{i}∥}_{\sum_{i}}^{2}.$$

In our system, UE poses represent the state vector as nodes and the constraints between successive poses as edges. The UE poses are modeled as NR5G factors and the edges of the graph are constraints, which are modeled as IMU and RSSI factors. The IMU factors are used to model the motion constraints through pre-integration and the RSSI factors correspond to the output of the BEB and serve as a correction pose.

The robot state vector x is modeled similarly to the factor graph formulation in [15], consisting of the rotation matrix ***R***∈SO(3), position ***p***$\in R^{3}$, velocity ***v*** vectors, and the IMU biases ***b***:

(4) $$x={\left[R^{T},p^{T},v^{T},b^{T}\right]}^{T}.$$

A transformation, T∈SE(3), from the robot base *B* to the world frame *W* is represented as T=[R|p].

**Figure 3:**
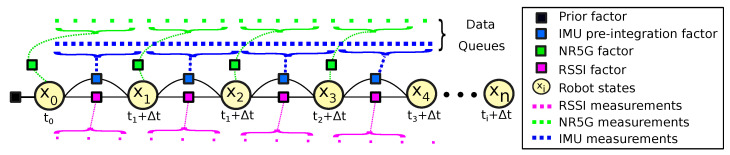
Overview of the NR5G-SAM factor graph consisting of the NR5G factors, IMU pre-integration factors, RSSI factor, and their individual data queues. A prior factor has been assigned to the first node of the graph at startup.

The output of the FEB prediction process is the full pose of the robot, namely the NR5G-SAM localization, consisting of position $\left[p_{x},p_{y},p_{z}\right]$, with the IMU orientation represented in quaternions $\left[q_{x},q_{y},q_{z},q_{w}\right]$ and pre-integrated linear and angular velocities $\left[v_{x},v_{y},v_{z},\omega_{x},\omega_{y},\omega_{z}\right]$. A description of the NR5G, IMU, and RSSI factors is presented below, and an illustration of the NR5G-SAM factor graph is shown in Figure 3.

#### 3.3.1. Prior Factor

A prior factor is required in Factor Graph SLAM to initialize the starting pose of the robot and to keep track of its states during the mapping process. It is a constraint that represents the known information about the robot’s pose or the landmark locations in the map.

The prior factor defines the initial values of the NR5G pose, RSSI pose, and IMU variables in the factor graph and constrains the subsequent observations based on these values. It contains the prior belief about the robot’s initial pose and/or the known locations of certain landmarks in the environment. It plays a crucial role as without the prior factor, the optimization process would not be properly initialized and the solution could converge to a wrong estimate.

#### 3.3.2. NR5G Factors

We have introduced a NR5G factor to receive absolute position measurements and velocity from a terrestrial positioning method similar to GNSS. As the factor graph is constructed, we associate new NR5G factors for each additional position $p_{i}$ within the state vector $x_{i}$. Measurements are stored in data queues to compensate for differences in the frequency update rates of the sensors. The NR5G factor is used as a node in the factor graph to model the relationship between the robot’s position and speed over time.

#### 3.3.3. IMU Factors

When new angular velocity and linear acceleration measurements are received from the onboard IMU, which is running at a high acquisition rate, we model them in our approach as [15,81]:

(5) $${\hat{\omega }}_{t}=\omega_{t}+b_{t}^{\omega }+n_{t}^{\omega }$$

(6) $${\hat{a}}_{t}=R_{t}^{\mathrm{BW}}\left(a_{t}-g\right)+b_{t}^{a}+n_{t}^{a},$$

where ${\hat{\omega }}_{t}$ is the raw angular velocity, computed from the current velocity $\omega_{t}$, the slowly varying bias $b_{t}^{\omega }$, and its respected noise $n_{t}^{\omega }$ at the given time step. The same applies for the raw acceleration ${\hat{a}}_{t}$, which is computed from the rotation matrix $R_{t}^{BW}$ between the base *B* of the robot and the world reference frame *W*, the current acceleration $a_{t}$, and the constant gravity vector g. The addition of a slowly varying bias $b_{t}^{a}$ and noise $n_{t}^{a}$ complete the equation.

As such, we are able to estimate the motion of the robot in the next timestep. This includes the position, velocity, and orientation, known as the IMU preintegration (see [81]), described by the following set of equations:

(7) $$v_{t+\Delta t}=v_{t}+g\Delta t+R_{t}\left({\hat{a}}_{t}-b_{t}^{a}-n_{t}^{a}\right)\Delta t$$

(8) $$p_{t+\Delta t}=p_{t}+v_{t}\Delta t+\frac{1}{2}g\Delta t^{2}+\frac{1}{2}R_{t}\left({\hat{a}}_{t}-b_{t}^{a}-n_{t}^{a}\right)\Delta t^{2}$$

(9) $$R_{t+\Delta t}=R_{t}\exp\left(\left({\hat{\omega }}_{t}-b_{t}^{\omega }-n_{t}^{\omega_{t}}\right)\Delta t\right),$$

where $v_{t+\Delta t}$ is the linear velocity, $p_{t+\Delta t}$ is the position, and $R_{t+\Delta t}$ the rotation matrix at the inertial frame *I*, which coincides with the robot base frame *B* for simplicity, at each timestep. Their overall differences over time are computed as follows:

(10) $$\Delta v_{ij}=R_{i}^{⊤}\left(v_{j}-v_{i}-g\Delta t_{ij}\right)$$

(11) $$\Delta p_{ij}=R_{i}^{⊤}\left(p_{j}-p_{i}-v_{i}\Delta t_{ij}-\frac{1}{2}g\Delta t_{ij}^{2}\right)$$

(12) $$\Delta R_{ij}=R_{i}^{⊤}R_{j}.$$

This process is suitable for real-time applications as the generated FEB pose is provided at a high rate (200 Hz in our setup, as shown in Section 4). The output of the Front-End Block is the NR5G-SAM Localization, which feeds the Back-End block.

#### 3.3.4. RSSI Factors

This factor is the output of the BEB block and consists of the estimated pose of the robot, based on the RSSI positioning generated from the RSSI measurements, that are obtained from the robot. It is introduced as an additional pose estimate generated from range free methodology compared to 5G positioning, which is the result of multilateration. It is an additional pose measurement to aid the prediction process of the FEB. A RSSI factor is added to the graph in place of the NR5G factor with the intent to correct the NR5G poses over time.

Upon reception of the RSSI factors, we transform them to the world coordinate frame W. When a new node is added to the factor graph, we associate it also with a new RSSI factor measurement. For the correction step, a new RSSI factor is only added in place of the NR5G factor, if the UE position covariance (coming from the UEB) is larger than the received RSSI position covariance coming from the BEB.

### 3.4. Back-End Block (BEB)

The BEB utilizes the generated real-time NR5G-SAM localization data from the FEB as an initial estimate for a GA optimization step. The GA then seeks to minimize the difference between the RSSI measured by the mobile robot and the estimated grid-based REM, generated using the UMa model. The RSSI vector consists of the individual power level measurements of each gNB and is formulated as Equation (14). It is then used as a reference for the GA to find the most suitable position within the global search space and within the bounds of radius $R_{s}$ around the FEB estimation.This approach generates RSSI positioning similar to other interpolation methods, such as inverse distance weighting (IDW). It is important to note that larger values of $R_{s}$ result in longer convergence times for the GA, which may lead to less frequent position estimates from the BEB. Conversely, a smaller $R_{s}$ increases the dependence on the NR5G-SAM localization data from the FEB, which operates at the IMU frequency.

**Figure 4:**
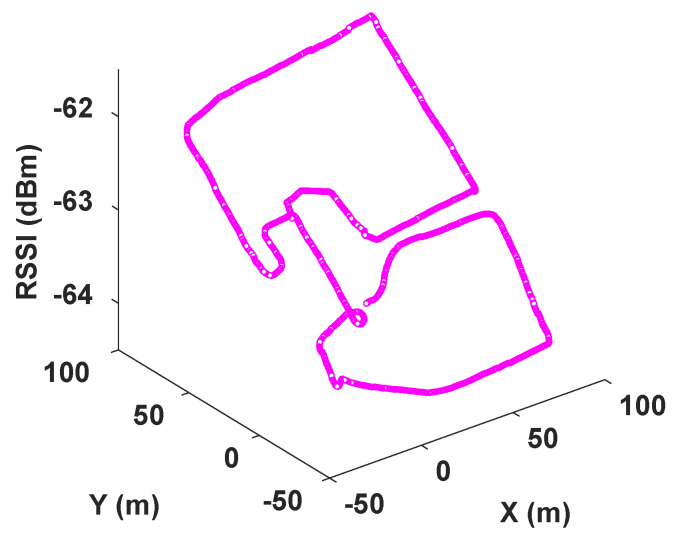
RSSI measurements for a gNodeB station along a robot’s trajectory. Higher RSSI values are measured when the robot is closer to the gNodeB station, as generated by the 5G NR path loss model.

Figure 4 exemplifies how RSSI measurements from a single gNoneB station vary while a robot traverses an environment, following the UMa path loss model. From both the RSSI measurements and the UMa model, we find the optimal position that minimizes error of the GA’s fitness function. Specifically, we adopt the following path loss model, as per Equation (13):

(13) $$\begin{array}{c} PL=28.0+22*log10\left(d_{r}\right)+20*log10\left(fc\right), \end{array}$$

for the LoS use case from [13], where $d_{r}$ is the estimated range generated based on the RSSI acquired by the robot for each gNB and $f_{c}$ is the carrier frequency in GHz set accordingly for the sub-6 GHz FR1 and the mmWave FR2 cases (see Section 4).

Moreover, the RSSI positioning is coupled with IMU and the wheel velocity measurements within an EKF, using the same configuration as the UEB. This is a necessary step due to the nature of the RSSI measurements varying and being sensitive to the environmental conditions. This process reduces noisy estimations of the RSSI positioning and fuses the information to obtain a complete mapping pose estimate.

In addition to providing a position estimation based on the RSSI values, the BEB is also responsible for performing a cellular 5G radio mapping, which is foreseen as a crucial component for the future implementation of a loop closure condition. This process involves mapping out the signal strength and coverage of a given area as the robot navigates. This is performed by storing the radio signal data of all available gNBs from different locations. These maps are commonly known as REMs and provide a representation of the signal power drop over the robot’s environment, represented as a bi-dimensional grid map of RSSI values for each gNB. The rem grid map is formulated as Equation (15).

The generated REMs are based on the 5G UMa model and represent the slopes of the received signal power indication levels paired along with a grid of positions in the surrounding area. The grid size can be set to a specific size, which in our case includes the wider application area including the gNB locations. A resolution threshold can be set according to a fixed distance ratio. A color coding scheme has been introduced for the grid map cells, which represents the degradation of the signal power due to propagation distance. The peaks observed represent the points where the base stations have maximum RSSI levels (i.e., where the gNBs are located). The following equations are the representation of the REMs based on the rss vector and the merged global map, which is indexed by position and has the world frame *W* as a common reference.

(14) $$\mathrm{rss}=\left[rss_{1},rss_{2},\ldots ,rss_{n}\right]$$

(15) $${\mathrm{rem}}_{i}^{W}=\sum_{i}^{k}\left[x_{i},y_{i},rss_{i}\right]$$

Figure 2 also presents a global 5G radio environmental map, which was created by overlaying the individual maps for each station. This global map can be computed as each local map is represented in the global frame of reference *W*. The global map comprises a vector of *k* values per cell, with each corresponding to an individual RSSI value measured per gNB. The format of Equation (16) of the global 5G map M can be utilized for inference frameworks and learning-based optimization approaches, which can leverage the map as a navigation aid and correct the robot’s localization.

(16) $$M=\sum_{i}^{n}\left[x_{i},y_{i},rem_{i:n}\right]$$

Such an approach can help the robot to proactively identify loop closure and, with it, correct errors in its estimated pose by identifying previously visited RSSI measures and re-observing them. This helps to reduce accumulated errors in the estimated robot pose over time and improve the accuracy of the map. It is noteworthy that, at the current stage of the work, we have not yet implemented loop closure within the proposed architecture, though it is foreseen as future work (see Section 7).

## 4. Experimental Validation

**Figure 5:**
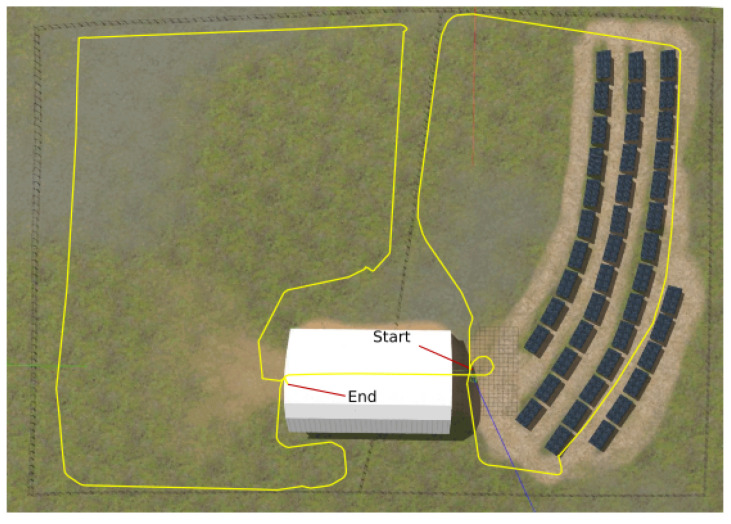
Ground truth trajectory of the robot (depicted in yellow) in the simulated agriculture environment.

In a previous work [16], we developed a realistic simulation testbed that includes an agricultural environment, as shown in Figure 5. We use the Robot Operating System (ROS) (https://www.ros.org, accessed on 4 March 2023) for perception and actuation of the field robot, and the Gazebo simulator (https://gazebosim.org, accessed on 4 March 2023) with integrated physics engine that generates the ground truth pose and the required raw data from the robot’s sensors, including the IMU and wheel odometry. The sensors are modeled with additional Gaussian noise, which is applied to the IMU and wheel odometry measurements. The simulation environment also accounts for elevation, gravity, and other physical properties, whereas the 5G NR radio propagation is generated separately.

We have coupled the simulation engine with the 5G MATLAB Toolbox (https://www.mathworks.com/products/5g.html, accessed on 4 March 2023) for the 5G NR configuration and signal transmission. A key fact about the rollout of 5G NR is that signal coverage determines the geometry of the gNB locations and is envisaged to be in a grid pattern in structured urban areas, with each station being approximately a kilometer apart from the distributed micro cells. In rural agricultural areas, the gNB deployment geometry may vary depending on the morphology and is envisaged to operate under macro cells. The path loss model used within our simulation is set to the UMa, with the infrastructure height set to 5 m, the environmental height set to 2 m, and being representative of a relatively flat agricultural area, similar to the Gazebo world. The height of the gNBs is set to 25 m and the height of the UE antenna is set to 1 m; it is modeled as an attachment on the robot centered above the base frame *B* with known offset. In our experiments, we assume that all stations of the cellular network are synchronized and have the ability to communicate network time information based on a common reference.

Two setups have been prepared, where we leverage the 5G NR PRS signals in the FR1 and FR2 frequency ranges. We selected the n78 band with a Sub-Carrier Spacing (SCS) of 60 KHz for the sub-6 GHz (FR1) and the n257 band with SCS 240 KHz for the mmWave (FR2). In the configuration of the 5G NR signal parameters, the PRS depends on the DL-ToA scheme and we configured the carrier slots of each gNB and a carrier frequency of 3.5 GHz for FR1 and 29.5 GHz for FR2. The transmission offset was set to be every one consecutive repeated PRS signal. The modulation scheme for the Physical Downlink Shared Channel (PDSCH) is set to Quadrature Phase Shift Keying (QPSK) and the length of the codewords to be 2 bits per symbol. The cyclic prefix is set to normal, which corresponds to 14 OFDM symbols within a slot. Orthogonal frequency-division multiplexing modulation OFDM is then performed to generate the 5G NR waveforms at each gNB for transmission. The hearability problem [82] is addressed by allocating the PRS resources and PDSCH channel to the slot grid in such a way that no other gNB transmits a signal within the same slot. As a last step, they are received by the robot and extracted by the UEB (as shown in Section 3.2).

**Figure 6:**
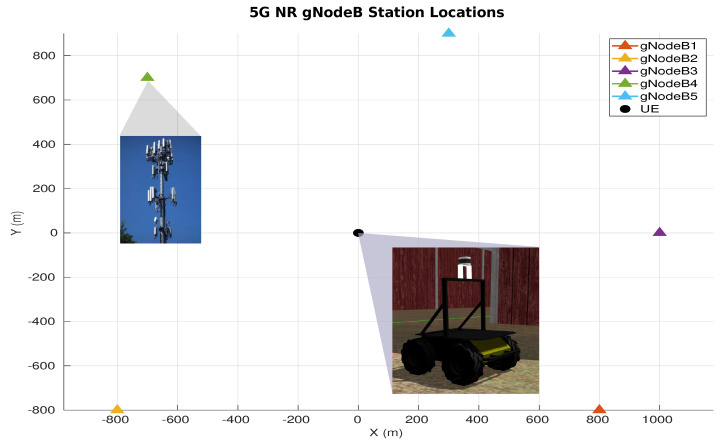
Base stations locations for the experiment using 5G NR Macro cells and the starting location of UE that is attached to the robot base.

The simulated robot is a Husky Unmanned Ground Vehicle (UGV) from Clearpath Robotics (https://clearpathrobotics.com/husky-unmanned-ground-vehicle-robot, accessed on 5 March 2023). In this work, we set the number of gNB stations to n=5 with their fixed locations as the following list of coordinates in the world frame: s1 = [800,−800], s2 = [−800,−800], s3 = [1000, 0], *s*4 = [−700, 700], and s5 = [300, 900], as illustrated in Figure 6. The starting point of the robot is the origin [0,0] position.

To allow for a comparative evaluation of methods, we have driven the robot within the environment, as shown in Figure 5, while recording all of the necessary signals and raw data, including the ToA information, which is converted to a position within the UEB, along with the associated CSI measurements. We also extract the ground truth trajectory of the mobile robot from the simulator, which we use only for evaluating the localization accuracy of the estimation methods compared.

Note that the right-hand side of the environment consists of inclined solar panels mounted on the ground inside the fenced area. On the left-hand side, there is an empty area that is surrounded only by wooden fences, which delimits the two sides of the environment. The transition between the two areas is made through an empty barn structure, which is not modeled with signal reflection or obstruction properties, as it is out of our current scope. The overall dimensions of the environment are 120×90 m.

In terms of positioning, the GA optimization of the UEB follows the setup presented in [16]. For the BEB, the GA has been set with a population size of 200 and an elite count of 0.05. The maximum iterations has been set to 20. Regarding the EKF setups, we provide IMU and wheel velocities from the onboard sensors along with the 5G and RSSI positioning to generate the pose estimates for each block using the implementation described in [79], which is a well-established ROS-based localization framework in the robotics community.

We compare our proposed method with LIO-SAM (https://github.com/TixiaoShan/LIO-SAM, accessed on 7 March 2023), a LiDAR-Inertial SLAM approach [15], in order to demonstrate its capabilities. The reasoning for this decision is that it is a widely used state-of-the-art factor graph-based approach that utilizes GTSAM (https://github.com/borglab/gtsam, accessed on 7 March 2023), a C++ factor graph optimization library developed by Dellaert et al. [83], for real-time pose estimation and mapping. A LiDAR sensor modeled after the Velodyne VLP-16 (https://velodynelidar.com/products/puck/, accessed on 7 March 2023) is added to our robot to be used for localization and mapping of the environment with LIO-SAM. The parametric setup of the algorithm is set to the default and we followed the author’s guidelines to set it up for outdoor environments.

We draw similarities in the modeling of NR5G-SAM as we are utilizing the GTSAM library for smoothing and mapping, with the difference that we developed a cellular radio-inertial pose estimation and mapping method. Regarding the sensor acquisition rates, the IMU provides data at 200 Hz and the wheel odometry is communicated at 50 Hz. The 5G positioning publishes at the rate of 25 Hz and is representative for the sub-6 GHz FR1 setup. For the mmWave FR2 setup, the publication rate is 35 Hz, which is faster as we are transmitting in the mmWave frequency. The NR5G-SAM localization is published at the IMU frequency and the mapping pose is published at 3 Hz due to the heavy GA optimization over each REM. The evaluation metrics follow next.

### Evaluation Metrics

For the localization performance assessment, we analyze the pose estimation of the NR5G-SAM method against LIO-SAM with the average RMSE metric. This metric measures the average deviation of the estimated positions from the ground truth and is expressed by:

(17) $$RMSE=\sqrt{\frac{1}{n}\sum_{i=1}^{n}{\left(x_{x,y}^{\left(i\right)}-\hat{x_{x,y}^{\left(i\right)}}\right)}^{2}},$$

where $x_{x,y}^{\left(i\right)}=\left(x_{i},y_{i}\right)$ are the ground truth positions and $\hat{x_{x,y}^{\left(i\right)}}=\left(\hat{x_{i}},\hat{y_{i}}\right)$ are the estimated positions.

We perform the evaluation considering the 2D pose estimation in our experiments due to the large z error, which is associated with multilateration in radio-based positioning. The reason being that the accuracy of the estimated position is affected by several factors, most importantly by the number and distribution of gNBs, the accuracy of their estimated position, and the measurement noise. The latter is proportional to the signal propagation time as the speed of light is constant and the measurement noise is proportional to the distance traveled by the signal, which is larger in the z-axis than in the x- and y-axes and was identified during our experiments. Additionally, the genetic algorithm may converge to sub-optimal solutions if the noise in the measurements is too large, which can subsequently lead to erroneous z estimates on the z-axis and has been identified through design steps of the proposed method. In the future, we consider benefiting from sensor fusion with other sensors, such as an altimeter, to improve the accuracy of the z estimates in real-world experiments.

As a second metric, the standard deviation of the errors for each estimated trajectory is given by Equation (18). This represents the expected deviation between the expected error for the estimations performed by the 5G positioning, NR5G-SAM and LIO-SAM approaches, and the ground truth.

(18) $$Sn_{\left(x,y\right)}=\sqrt{\frac{1}{n}\sum_{i=1}^{n}{\left(x_{i}-\mu_{x}\right)}^{2}{\left(y_{i}-\mu_{y}\right)}^{2}}$$

Regarding the mapping process, we propose REM as an initial representation of how the signal fades along the environment for qualitative evaluation. The multi-layer grid map REM provides a comprehensive representation of the radio signal environment and allows the robot to not only identify areas with strong and weak signal strengths, but also provide a unique RSSI feature vector for each position. It is noteworthy that, in this work, the RSSI vector played a key role in improving the robot pose estimation provided by the 5G NR-based approach, even though the REM formed by such a vector over time was not fully explored. Nonetheless, the qualitative assessment of the REMs offered here intends to emphasize the use of the BEB as a critical loop closure component of the proposed architecture.

## 5. Results and Discussion

In this section, we discuss the results obtained using the proposed 5G SLAM method in the delineated experimental scenarios. In general, we consider the performance of NR5G-SAM as a standalone radio-inertial SLAM approach to be an attractive choice for adaptation within field robot applications. In more detail, we analyze the performance of the method in the following paragraphs.

**Figure 7:**
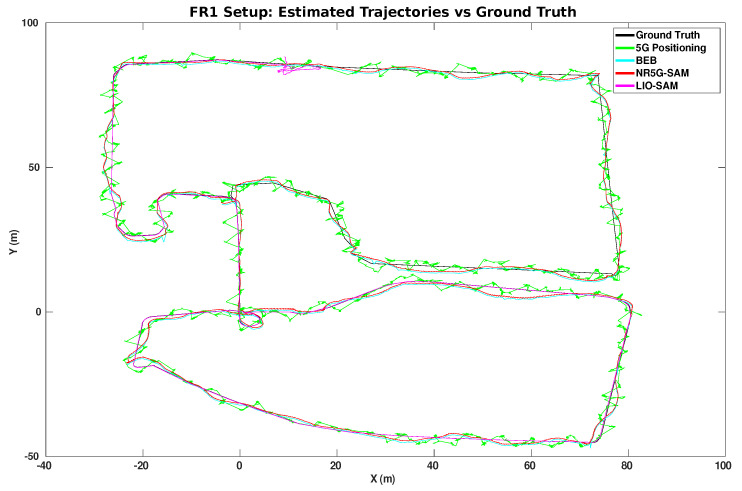
The trajectories performed under the FR1 Setup using sub-6 GHz frequencies.

Overall, NR5G-SAM performed adequately in the FR1 setup by scoring sub-meter accuracy on average (as shown in Table 1), while traversing the environment. The setup used a transmission frequency of 3.5 GHz and sub-carrier spacing of 60 kHz, which is a band used already by smartphones. The overall trajectories performed can be seen in Figure 7, where the 5G positioning from the UEB can be seen as a noisy trajectory in green, due to weak signals arriving from gNBs, which are at a distance. The estimated trajectories of the FEB in red and the BEB in cyan for this use case can be seen as smoothed trajectories meeting the half meter error mark on average overall.

**Figure 8:**
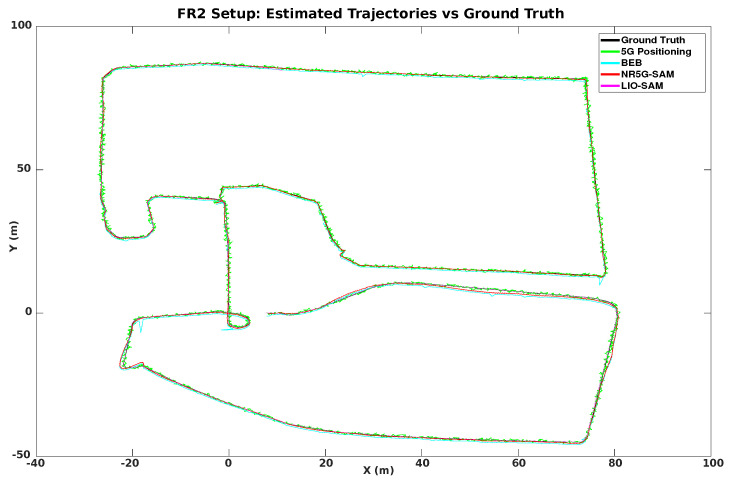
The trajectories performed under the FR2 Setup using mmWave frequencies.

As we increased the sub-carrier spacing to 240 kHz and the transmission frequency to 29.5 GHz, we experienced the advertised benefits of mmWave as a large improvement in the 5G positioning, which is reflected by the results of Table 2 and the demonstrated trajectories of Figure 8. The estimated trajectories demonstrate a great potential in approaching the ground truth performance, with error results similar to other works [33].

**Figure 9:**
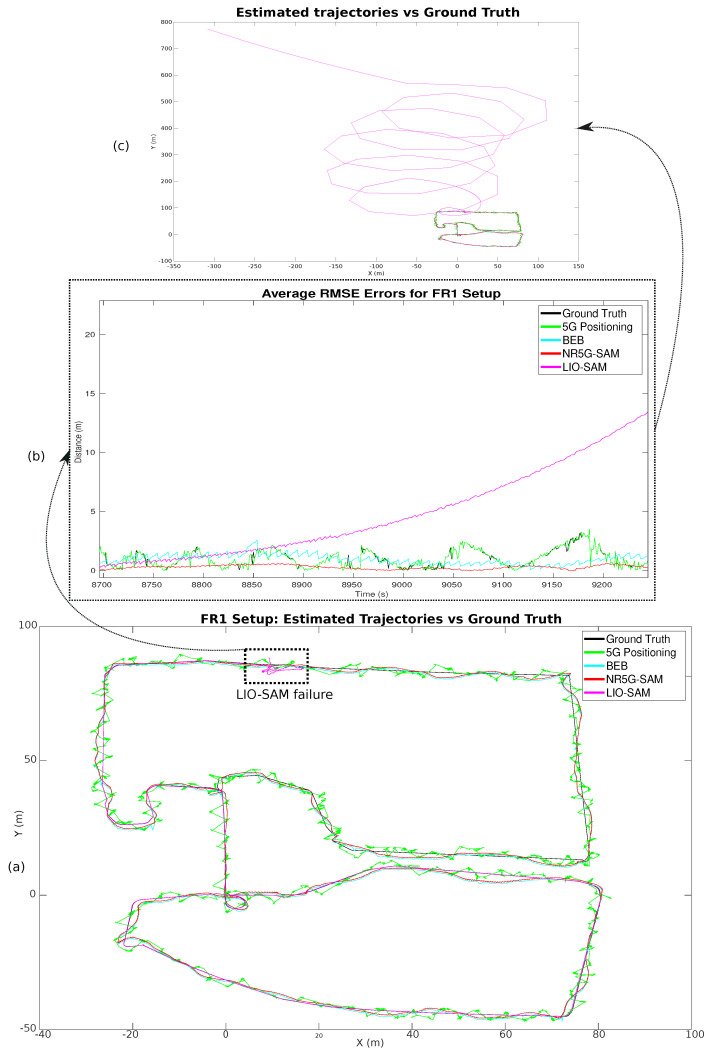
(**a**) Overall estimated trajectories, including a highlight of the (**b**) average RMSE error distributions, with emphasis on the failure moment and (**c**) LIO-SAM getting lost due to the lack of features; we maintain localization from NR5G-SAM.

**Figure 10:**
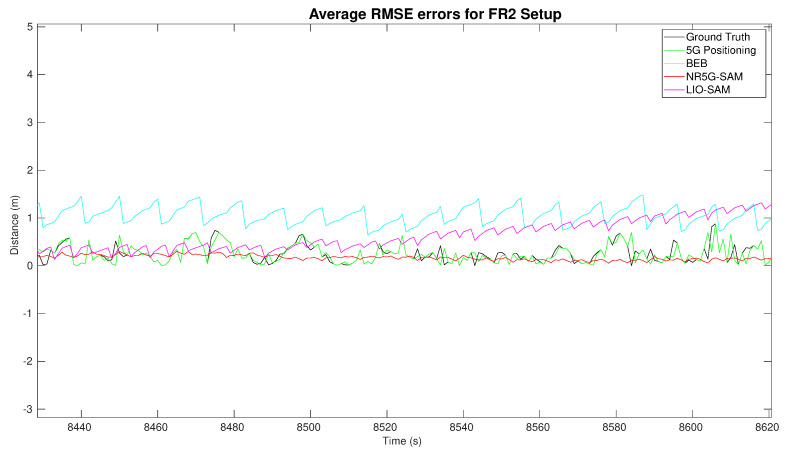
Error distributions of the estimated trajectories for the FR2 setup at the point of LIO-SAM failure.

A noteworthy result is that LIO-SAM failed to estimate the localization for the full robot trajectory due to the lack of features and the homogeneity of the environment. A highlight of the error distributions can be seen in Figure 9b for FR1 and Figure 10 for the FR2 setup with an emphasis on the moment of failure for LIO-SAM. It can be observed that the error increases quadratically. This did not occur during the right half of the trajectory (as shown in Figure 5) due to having more features to track, such as the solar panel edges and planes. This is a common disadvantage of LiDAR-based SLAM approaches in outdoor environments such as rural areas, either due to the similarity of features or the lack of them. On the other end, NR5G-SAM presents immunity against these type of errors and is able to support the robot throughout the whole experiment, as is evident in the generated trajectories as it is a radio-based approach.

In terms of the localization performance, the comparison considers the 5G positioning from the User Equipment Block of our approach and the results from the NR5G-SAM framework, as well as LIO-SAM. We have measured the average RMSE for the FR1 setup (Table 1) and the FR2 setup (Table 2) by considering both cases: prior and post LIO-SAM failure.

In the FR1 setup and prior to LIO-SAM failure, the error for 5G positioning is 0.90 m and NR5G-SAM presents 0.55 m with a standard deviation of ±0.47 m, which is an improvement from our previous work [16], as we have utilized an EKF for filtering the large deviations. LIO-SAM performed with an overall error of 0.12 m compared to the ground truth with a standard deviation of ±0.26 m, demonstrating the huge potential of LiDAR SLAM approaches in favorable scenarios. Despite underperforming, NR5G-SAM preserved the localization of the robot bounded, during the post failure case and until the end of the robot’s trajectory by scoring a RMSE of 0.52 m with a standard deviation of ±0.46 m, compared to LIO-SAM with 52.87 m and a standard deviation of ±106.84 m. The results of the 5G positioning were similar for the prior case at 0.91 m on average, with a standard deviation of ±0.65 m. The estimated trajectories for this setup can be seen on the top of Figure 7.

In the FR2 setup and prior to LIO-SAM’s failure, 5G positioning presents a RMSE of 0.22 m, with a standard deviation of ±0.16 m. NR5G-SAM presents a RMSE of 0.23 m and LIO-SAM yields a RMSE of 0.12 m, with a standard deviation of ±0.26 m. This significant increase in the accuracy of NR5G-SAM is due to the numerology features of the the 5G NR mmWave and the minimization in the sub-carrier spacing of the slots, hence transmitted frames of the signal. For the post-failure case, the 5G positioning presents the same RMSE with 0.22 m and a standard deviation of ±0.16 m. NR5G-SAM shows an error of 0.19 m, with a standard deviation of ±0.19 m. This increase in performance is due to the mobility of the robot performing linear trajectories, instead of curved ones and having less rotations, as opposed to the area of the environment prior to LIO-SAM’s failure (as shown in Figure 5). Again, LIO-SAM was not able to support the localization for the previously mentioned reasons and, hence, provided an error of 53.61 m, with a standard deviation of ±107.49 m. The estimated trajectories for the FR2 setup can be seen on the bottom of Figure 8.

**Figure 11:**
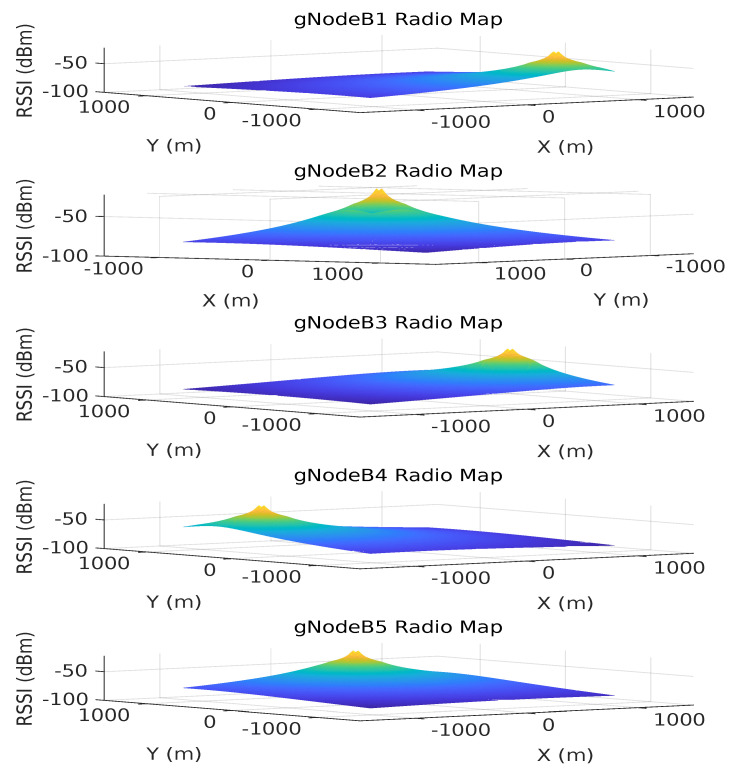
Individual Radio Environmental Maps created from the UMa Path Loss model using five gNB stations.

**Figure 12:**
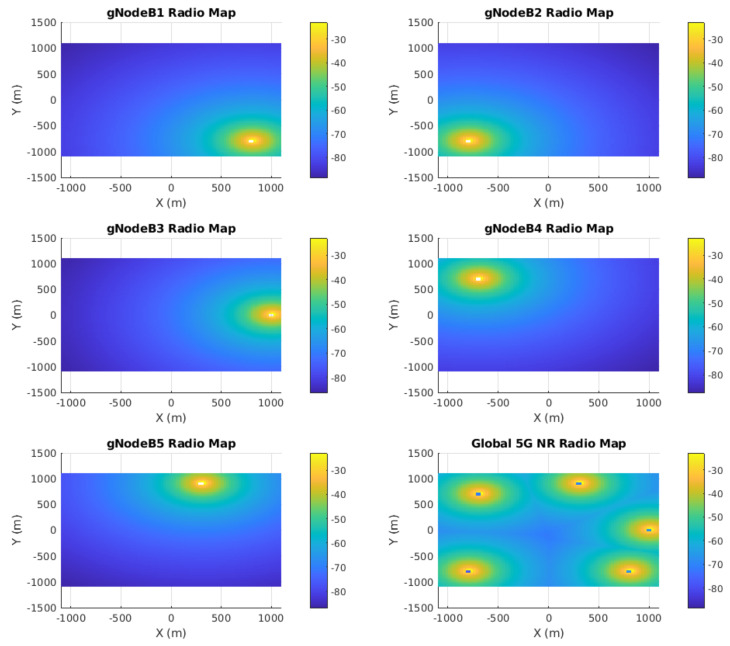
Individual REMs for FR2 setup along with the 2D global 5G REM.

As the result of the radio mapping process, individual maps have been generated and can be seen in Figure 11 and their 2D representation in Figure 12. Each map consists of the RSSI measurements, expanded to the additional cells of the grid using the UMa propagation equations. The measured signal strength is measured along the recorded trajectory. We observe in each individual map that values tend to increase as we approach nearer to a gNB station, which can provide us with proximity information and, hence, can be used to estimate positioning in the global reference frame. We can estimate the position based on the comparison between the measured RSSI and the estimated one using the UMa model. As can be seen in Figure 9b and Figure 10, the cyan trajectory of the BEB poses is in general larger in error than the NR5G-SAM localization, but some measurements are more accurate over time, which we use for corrections. For the mapping pose estimates that have less covariance than the UE pose, we select the BEB pose estimate for position correction. During robot motion, we consider the pose as an additional factor in the graph.

**Figure 13:**
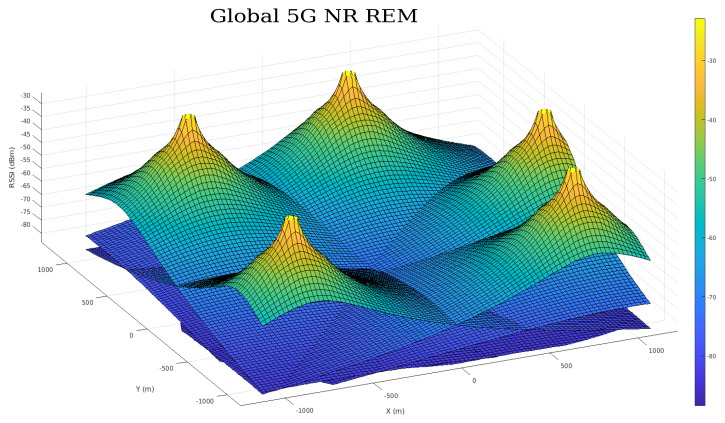
A 3D representation of the global 5G REM generated from the individual gNodeB radio maps. Peaks represent the gNodeB locations and the color highlights the signal strength degradation throughout the environment.

The global 5G REM is the result of merging the individual $\left[rem_{1},rem_{2},\ldots ,rem_{n}\right]$ maps. In the current stage of our work, we provide a representation of a unified grid map representation (as shown in Figure 13) consisting of an n=5 sized vector for each cell. A clear advantage that can be gained from the radio mapping process is the employment of a loop closure mechanism in order to provide scene recognition capability within the radio environment. We believe that this is an important next step within the current methodology that can provide significant information gains for operational environments and correct the poses of the graph in places that have been revisited based on RSSI values.

Summing up, NR5G-SAM is a method of localization and mapping that uses radio signals instead of visual or laser sensors. It involves measuring the signal strength and time of arrival of 5G NR radio waves from a known location to a robot’s receiver. By comparing the characteristics of these waves to an estimated map of signal strengths, the robot can estimate its own pose relative to the map. This technique has the advantage of being able to work in environments where visual and laser sensors may be impeded. We foresee its potential in areas with limited GNSS coverage as it provides absolute measurements that are geographically referenced to nearby gNodeB stations in urban or rural scenarios.

## 6. Lessons Learned

The pose estimation capability of the UEB just based on 5G positioning requires filtering from an EKF or other optimization approach to minimize the large errors, which are introduced during signal reception. The signal quality and the physical interaction of the emitted signal with the environment is envisaged to be crucial to performance. An important factor during the early developments of the method is the geometry of the gNB stations. For our experiments, we made estimations for the robot inside the coverage area of the surrounding stations. In reality, with a mobile robot in a dynamic mobility scenario, this can have a detrimental effect on the localization unless mitigation actions are considered, such as automatic cell selection and switching to maintain the integrity of localization performance.

Signal reflections, scattering, and refractions occur in real applications, and interference from other sources will impact the uncertainty within the measurements as additional noise. We assumed that the noise is Gaussian, but in reality this may vary. However, we foresee that the growing availability of 5G NR in urban areas where it is currently widely available as SA or Non Stand Alone (NSA) and further research, will resolve some of these important issues.

Within this study, we show that it is possible to estimate the pose of the robot via sensor fusion with an EKF, along with inertial sensing and robot wheel velocities using 5G NR. We utilize the IMU as an orientation, angular velocity and acceleration source, as it is widely available as an onboard resource of mobile robotic systems. We have considered the LoS signals from gNBs, which is not necessarily a limitation, as there are rural and urban regions that have these qualities. Despite our choice for simplification, Non-Line-of-Sight (NLoS) is a foreseen problem and multi-path effects in the FR1 frequencies. In FR2, it has been shown in previous works that a multi-path signal can help in the pose estimation process within urban and indoor environments, as the emitted rays have a low penetration ability on structures and instead reflect off of them, hence can be used as VAs for ray-tracing approaches.

In terms of FEB, we have shown that the factor graph adoption within the proposed approach can provide a smoothed localization that is robust over time. The errors are handled efficiently and the predictions are fast at 200 Hz, providing a real-time performance. It has assisted us in creating a modular representation that is capable of modeling the relationship of the robot motion with the IMU factor, the robot pose from the NR5G factor and RSSI factor measurements, during the design phase of the overall SLAM problem. Additionally, it provided us with modularity and extendability of NR5G-SAM from the early design phase, which is highly attractive for maintaining and scaling the SLAM approach with additional sources as factors.

The BEB serves as an example of how signal metrics can assist the pose estimation process of a robot in outdoor environments. In our approach, we have used the RSSI values from each gNB station along with the UMa model to predict the position of the robot. We have chosen to use the RSSI as an experiment due to the wide adoption in UWB and WiFi approaches for position estimation [10]. The BEB pose estimates present, in general, more noisy behavior compared to multilateration output. Moreover, we chose this direction as it can provide a double benefit of generating REMs and a pose estimate for robot localization purposes. The accuracy can be further improved if more measurements are performed in the application area as in fingerprinting [61], which can be a laborious effort for early deployments of robots outdoors and online learning-based approaches may assist towards this direction by inferring patterns within the measurement variation over time [84].

In the case of poor 5G signal, the robot will rely on other sensing information in order to preserve the localization over time, similar to what has been presented in our current work as a comparison. For example, as LiDAR localization fails the NR5G-SAM is envisaged to assist as an absolute pose information source. The equivalent case in real scenarios is when GNSS fails and a robot is forced to rely locally on LiDAR or visual inertial localization for a given amount of time until the signal is recovered.

As a final mark, we have focused on LoS conditions and the consideration of potential infrastructure according to the 3GPP UMa model during the acquisition of 5G signals. The error sources that we have considered and mitigated are the (i) dynamic mobility error and measurement noise, (ii) the uneven distribution of the gNB stations and (iii) the interference from nearby gNBs, as a muting transmission scheme was ensured. Other sources that have not been included are: (i) NLoS conditions, (ii) obstructions, (iii) reflections, (iv) refractions, (v) scattering imposed from the surrounding obstacles of certain material characteristics and finally (vi) interference on the same frequency ranges and weather conditions, which remain future research topics.

## 7. Conclusions

In this work, we have presented the implementation of a preliminary cellular NR5G-SAM framework. We have evaluated its localization accuracy in terms of average RMSE and their standard deviations in two-dimensional Cartesian space for two radio setups in a simulated agricultural environment. We have also compared it to LIO-SAM to evaluate the robustness and overall performance. We have demonstrated that a cellular 5G SLAM can assist in localizing a robotic system in an outdoor setup with relatively low error margins in FR1 and FR2 frequencies. The overall accuracy was 0.52–0.55 m for sub-6 GHz and 0.19–0.23 m for the mmWave frequency, which is comparable to the performance of modern single receiver GNSS methods [22,85]. Due to the early stage of the work, we did not account yet for multi-path effects, including regions of non-line of sight (NLoS) use cases, which exist in real-world scenarios and will be addressed as part of our future work. Moreover, we presented an RSSI mapping representation process that can be further improved and used for the SLAM pose estimation correction process alongside the RSSI positioning.

NR5G-SAM can provide many benefits to the robotics communities as an additional pose information source in multi-modal sensor fusion approaches combined with GNSS, LiDAR, visual, and inertial sensing. In fact, LiDAR technology is already industrially mature compared to 5G NR sensing, but with time and further efforts it should be able to surpass GNSS in areas with coverage (e.g., see [22]) and support robotics applications in GNSS-denied environments.

An open question which inevitably falls into the scope of our future research is the comparison of NR5G-SAM under a real-world scenario against GNSS and its variants, as it would provide informative results compared to the global standard in outdoor positioning.

As a future development stage, we also intend to explore the estimation of orientation from AoA and AoD approaches, the reason being that 5G NR introduces the concept of beam-forming where each signal can be traced to the base station that emitted it, revealing the azimuth angle of transmission. Also, the angle of reception can be estimated from the robot with an antenna array. Regarding velocities, similar to GNSS systems, the estimation of motion can be performed via the Doppler effect [86], which can be used as a reference to minimize uncertainty, and the UEB RSSI variability can be learned with machine learning methods [87,88], having the potential to be included and adopted within our framework.

Moreover, we foresee the integration of the complete CSI parameters such as the Reference Signal Received Power (RSRP) and Reference Signal Received Quality (RSRQ), which are often used for cellular signal diagnostics, in the optimization process and including them as factors in the factor graph. We also intend to introduce a loop closure constraint within NR5G-SAM based on the complete set of signal propagation parameters and REMs to further improve the localization performance of the framework. With this additional capability, we aim to tackle multi-path effects and even take advantage of them for localization as unique NLoS features.

Furthermore, a macroscopic ambition would be to combine spatial maps acquired by LiDAR sensors with REMs to enable enhanced localization and scene understanding capabilities in outdoor environments that can assist in mapping GNSS-denied environments, such as indoors, urban, and other rural areas (e.g., forests, mines). Effective communication will be an important challenge for robotic systems operating in outdoor complex environments to support localization capabilities.

All in all, we intend to perform an analysis of the method’s robustness under an urban scenario and with degraded cellular signals, which would assess the performance of the algorithm under various levels of 5G signal quality. This analysis is of the utmost importance and can help us identify the point at which the algorithm’s performance starts to degrade and to evaluate how gracefully it degrades over time. Due to its importance and real-world requirements, we expect to fulfil; this study with real 5G-enabled robots rather than in simulation. Finally, we plan to extend the NR5G-SAM approach to multi-robot systems in challenging real-world urban and rural environments. 

## Figures and Tables

**Table 1:** FR1 results for the estimated trajectories for 5G Positioning, NR5G-SAM, and LIO-SAM, including the average Root Mean Squared Errors and their Standard deviations for the cases of prior and post LIO-SAM failure (in meters).

Case	5G Positioning	NR5G-SAM	LIO-SAM
Prior	0.90±0.66	0.55±0.47	0.12±0.26
Post	0.91±0.65	0.52±0.46	52.87±106.84

**Table 2:** FR2 results for the estimated trajectories for 5G Positioning, NR5G-SAM, and LIO-SAM, including the average RMSEs and their standard deviations for the cases of prior and post LIO-SAM failure (in meters).

Case	5G Positioning	NR5G-SAM	LIO-SAM
Prior	0.22±0.16	0.23±0.22	0.12±0.26
Post	0.22±0.16	0.19±0.19	52.87±106.84

## Data Availability

The data presented in this study are available on request from the first author.

## Abbreviations

The following abbreviations are used in this article:

3GPP	Third Generation Partnership Project
5G	Fifth Generation Mobile Network
ACF	Auto Correlation Function
A/D	Analog-to-Digital
AoA	Angle of Arrival
AoD	Angle of Departure
AP	Access Point
BEB	Back-End Block
BLE	Bluetooth Low Energy
BS	Base Station
BP	Back Propagation
CAV	Connected Autonomous Vehicle
CLP	Collaborative Localization Protocol
CRLB	Cramér–Rao Lower Bound
CSI	Channel State Information
CSI-RS	Channel State Information Reference Signal
DoA	Direction of Arrival
DoF	Degrees of Freedom
dBm	Decibel Milliwatts
DL	Down-Link
ESPRIT	Estimation of Signal Parameters by Rotational Invariance Technique
EKF	Extended Kalman Filter
FEB	Front-End Block
FFT	Fast Fourier Transform
FIM	Fisher Information Matrix
GA	Genetic Algorithm
GGMR	Growing Gaussian Mixture Regression
GNSS	Global Navigation Satellite Systems
gNB	gNodeB
IDW	Inverse Distance Weighted
IMU	Inertial Measurement Unit
IoT	Internet of Things
LS	Least Squares
LiDAR	Light Detection And Ranging
LoS	Line-of-Sight
LoRa	Long-Range Protocol
LTE	Long-Term Evolution
MAP	Maximum A Posteriori
MCS	Modulation Carrier Scheme
MEC	Mobile Edge Computing
MIMO	Multiple-Input Multiple-Output
MLE	Maximum Likelihood Estimator
mmWave	Millimeter Wave
MPC	Multi-Path Components
MLP	Multi-Layer Perceptron
NLoS	Non-Line-of-Sight
NSA	Non Stand Alone
NR	New Radio
OEB	Orientation Error Bound
OFDM	Orthogonal Frequency-Division Multiplexing
PEB	Position Error Bound
PF	Particle Filter
PRS	Position Reference Signal
PHY	Physical Layer
PDSCH	Physical Downlink Shared Channel
PRS	Positioning Reference Signal
QPSK	Quadrature Phase Shift Keying
RF	Radio Frequency
RBPF	Rao-Blackwellized Particle Filter
RLP	Round-Trip Localization Protocol
RMSE	Root Mean Square Error
RSRP	Reference Signal Received Power
RSRQ	Reference Signal Received Quality
RSS	Received Signal Strength
RSSI	Received Signal Strength Indicator
REM	Radio Environmental Map
ROS	Robot Operating System
RIS	Reconfigurable Intelligent Surfaces
SA	Stand Alone
SAM	Smoothing and Mapping
SLAM	Simultaneous Localization and Mapping
SNR	Signal to Noise Ratio
SLAM	Simultaneous Localization and Mapping
SCS	Sub-Carrier Spacing
ToF	Time of Flight
TDoA	Time Difference of Arrival
ToA	Time of Arrival
TWL	Two-Way Localization
UAVs	Unmanned Aerial Vehicles
UDN	Ultra-Dense Network
UE	User Equipment
UEB	User Equipment Block
UWB	Ultra Wideband
UMa	Urban Macro-Cell
UL	Up-Link
UGV	Unmanned Ground Vehicle
VA	Virtual Anchor
WiFi	Wireless Fidelity

## References

[1] Durrant-Whyte H., Bailey T. (2006). Simultaneous localization and mapping: Part I. IEEE Robot. Autom. Mag..

[2] Bailey T., Durrant-Whyte H. (2006). Simultaneous localization and mapping (SLAM): Part II. IEEE Robot. Autom. Mag..

[3] Khan M.U., Zaidi S.A.A., Ishtiaq A., Bukhari S.U.R., Samer S., Farman A. A Comparative Survey of LiDAR-SLAM and LiDAR based Sensor Technologies. Proceedings of the Mohammad Ali Jinnah University International Conference on Computing (MAJICC).

[4] Lategahn H., Geiger A., Kitt B. Visual SLAM for autonomous ground vehicles. Proceedings of the 2011 IEEE International Conference on Robotics and Automation.

[5] Zhao S., Zhang H., Wang P., Nogueira L., Scherer S. Super Odometry: IMU-centric LiDAR-Visual-Inertial Estimator for Challenging Environments. Proceedings of the 2021 IEEE/RSJ International Conference on Intelligent Robots and Systems (IROS).

[6] Xhafa A., del Peral-Rosado J.A., López-Salcedo J.A., Seco-Granados G. (2022). Evaluation of 5G Positioning Performance Based on UTDoA, AoA and Base-Station Selective Exclusion. Sensors.

[7] Wu X., Xiao B., Wu C., Guo Y., Li L. (2022). Factor graph based navigation and positioning for control system design: A review. Chin. J. Aeronaut..

[8] Kaess M., Ranganathan A., Dellaert F. (2008). iSAM: Incremental Smoothing and Mapping. IEEE Trans. Robot..

[9] Kaess M., Johannsson H., Roberts R., Ila V., Leonard J.J., Dellaert F. (2012). iSAM2: Incremental smoothing and mapping using the Bayes tree. Int. J. Robot. Res..

[10] Oguejiofor O., Okorogu V., Abe A., Bo O. (2013). Outdoor Localization System Using RSSI Measurement of Wireless Sensor Network. Int. J. Innov. Technol. Explor. Eng..

[11] Dinh-Van N., Nashashibi F., Thanh-Huong N., Castelli E. Indoor Intelligent Vehicle localization using WiFi received signal strength indicator. Proceedings of the 2017 IEEE MTT-S International Conference on Microwaves for Intelligent Mobility (ICMIM).

[12] Yang S.W., Yang S.X., Yang L. Method of improving WiFi SLAM based on spatial and temporal coherence. Proceedings of the 2014 IEEE International Conference on Robotics and Automation (ICRA).

[13] ETSI (2020). Study on Channel Model for Frequencies from 0.5 to 100 GHz (3GPP TR 38.901 Version 16.1.0 Release 16 (V16.1.0)).

[14] Zhang K., Zhang R., Wu J., Jiang Y., Tang X. Measurement and Modeling of Path Loss and Channel Capacity Analysis for 5G UMa Scenario. Proceedings of the 11th International Conference on Wireless Communications and Signal Processing (WCSP).

[15] Shan T., Englot B., Meyers D., Wang W., Ratti C., Rus D. LIO-SAM: Tightly-coupled Lidar Inertial Odometry via Smoothing and Mapping. Proceedings of the IEEE/RSJ International Conference on Intelligent Robots and Systems (IROS).

[16] Karfakis P.T., Couceiro M.S., Portugal D., Antunes C.H. A Comparative Study of Mobile Robot Positioning Using 5G-NR. Proceedings of the IEEE International Conference on Robotics and Automation (ICRA) Workshop in Innovation in Forestry Robotics: Research and Industry Adoption.

[17] Sato A., Nakajima M., Kohtake N. Rapid BLE Beacon Localization with Range-Only EKF-SLAM Using Beacon Interval Constraint. Proceedings of the 2019 International Conference on Indoor Positioning and Indoor Navigation (IPIN).

[18] Wang J., Meng Z., Wang L. (2021). Efficient Probabilistic Approach to Range-Only SLAM With a Novel Likelihood Model. IEEE Trans. Instrum. Meas..

[19] Huang J., Millman D., Quigley M., Stavens D., Thrun S., Aggarwal A. Efficient, generalized indoor WiFi GraphSLAM. Proceedings of the 2011 IEEE International Conference on Robotics and Automation.

[20] Kwasme H., Ekin S. (2019). RSSI-Based Localization Using LoRaWAN Technology. IEEE Access.

[21] Margolies R., Becker R., Byers S., Deb S., Jana R., Urbanek S., Volinsky C. Can you find me now? Evaluation of network-based localization in a 4G LTE network. Proceedings of the IEEE INFOCOM 2017—IEEE Conference on Computer Communications.

[22] Gante J., Sousa L., Falcao G. (2020). Dethroning GPS: Low-Power Accurate 5G Positioning Systems Using Machine Learning. IEEE J. Emerg. Sel. Top. Circuits Syst..

[23] Shah B.M., Murtaza M., Raza M. Comparison of 4G and 5G Cellular Network Architecture and Proposing of 6G, a new era of AI. Proceedings of the 5th International Conference on Innovative Technologies in Intelligent Systems and Industrial Applications (CITISIA).

[24] Laoudias C., Moreira A., Kim S., Lee S., Wirola L., Fischione C. (2018). A Survey of Enabling Technologies for Network Localization, Tracking, and Navigation. IEEE Commun. Surv. Tutor..

[25] Sivasakthiselvan S., Nagarajan V. Localization Techniques of Wireless Sensor Networks: A Review. Proceedings of the 2020 International Conference on Communication and Signal Processing (ICCSP).

[26] del Peral-Rosado J.A., Raulefs R., López-Salcedo J.A., Seco-Granados G. (2018). Survey of Cellular Mobile Radio Localization Methods: From 1G to 5G. IEEE Commun. Surv. Tutor..

[27] Esposti V.D. Ray tracing: Techniques, applications and prospect. Proceedings of the 2020 International Symposium on Antennas and Propagation (ISAP).

[28] Chaloupka Z. (2017). Technology and Standardization Gaps for High Accuracy Positioning in 5g. IEEE Commun. Stand. Mag..

[29] Witrisal K., Meissner P., Leitinger E., Shen Y., Gustafson C., Tufvesson F., Haneda K., Dardari D., Molisch A.F., Conti A. (2016). High-Accuracy Localization for Assisted Living: 5G systems will turn multipath channels from foe to friend. IEEE Signal Process. Mag..

[30] Koivisto M., Costa M., Werner J., Heiska K., Talvitie J., Leppänen K., Koivunen V., Valkama M. (2017). Joint Device Positioning and Clock Synchronization in 5G Ultra-Dense Networks. IEEE Trans. Wirel. Commun..

[31] Abu-Shaban Z., Zhou X., Abhayapala T., Seco-Granados G., Wymeersch H. (2018). Error Bounds for Uplink and Downlink 3D Localization in 5G Millimeter Wave Systems. IEEE Trans. Wirel. Commun..

[32] Abu-Shaban Z., Wymeersch H., Abhayapala T., Seco-Granados G. (2020). Single-Anchor Two-Way Localization Bounds for 5G mmWave Systems. IEEE Trans. Veh. Technol..

[33] Zeng X., Zhang F., Wang B., Liu K.J.R. (2021). Massive MIMO for High-Accuracy Target Localization and Tracking. IEEE Internet Things J..

[34] Wu Z.H., Han Y., Chen Y., Liu K.J.R. (2015). A Time-Reversal Paradigm for Indoor Positioning System. IEEE Trans. Veh. Technol..

[35] Zhang F., Chen C., Wang B., Lai H.Q., Han Y., Liu K.J.R. (2018). WiBall: A Time-Reversal Focusing Ball Method for Decimeter-Accuracy Indoor Tracking. IEEE Internet Things J..

[36] Pesko M., Javornik T., Košir A., Štular M., Mohorčič M. (2014). Radio Environment Maps: The Survey of Construction Methods. KSII Trans. Internet Inf. Syst..

[37] Denkovski D., Atanasovski V., Gavrilovska L., Riihijårvi J., Måhønen P. Reliability of a Radio Environment Map: Case of Spatial Interpolation Techniques. Proceedings of the 7th IEEE International ICST Conference on Cognitive Radio Oriented Wireless Networks and Communications (CROWNCOM).

[38] Alaya-Feki A.B.H., Jemaa S.B., Sayrac B., Houze P., Moulines E. Informed spectrum usage in cognitive radio networks: Interference cartography. Proceedings of the 19th IEEE International Symposium on Personal, Indoor and Mobile Radio Communications.

[39] Phillips C., Ton M., Sicker D., Grunwald D. Practical radio environment mapping with geostatistics. Proceedings of the 2012 IEEE International Symposium on Dynamic Spectrum Access Networks.

[40] Angjelicinoski M., Atanasovski V., Gavrilovska L. Comparative analysis of spatial interpolation methods for creating radio environment maps. Proceedings of the 2011 19th Telecommunications Forum (TELFOR) Proceedings of Papers.

[41] Yilmaz H.B., Tugcu T. (2015). Location estimation-based radio environment map construction in fading channels. Wirel. Commun. Mob. Comput..

[42] Sun G., van de Beek J. Simple distributed interference source localization for radio environment mapping. Proceedings of the IFIP Wireless Days.

[43] Ould Isselmou Y., Wackernagel H., Tabbara W., Wiart J. Geostatistical interpolation for mapping radio-electric exposure levels. Proceedings of the 2006 First European Conference on Antennas and Propagation.

[44] Barneto C.B., Riihonen T., Turunen M., Koivisto M., Talvitie J., Valkama M. Radio-based sensing and environment mapping in millimeter-wave 5G and beyond networks. Proceedings of the IEEE International Conference on Localization and GNSS (ICL-GNSS).

[45] Zhang J., Yu X., Letaief K.B. (2020). Hybrid Beamforming for 5G and Beyond Millimeter-Wave Systems: A Holistic View. IEEE Open J. Commun. Soc..

[46] Azpilicueta L., Lopez-Iturri P., Zuñiga-Mejia J., Celaya-Echarri M., Rodríguez-Corbo F.A., Vargas-Rosales C., Aguirre E., Michelson D.G., Falcone F. (2020). Fifth-generation (5G) mmWave spatial channel characterization for urban environments’ system analysis. Sensors.

[47] Heimann K., Tiemann J., Yolchyan D., Wietfeld C. Experimental 5G mmWave Beam Tracking Testbed for Evaluation of Vehicular Communications. Proceedings of the 2019 IEEE 2nd 5G World Forum (5GWF).

[48] Kim H., Wymeersch H., Garcia N., Seco-Granados G., Kim S. 5G mmWave Vehicular Tracking. Proceedings of the 2018 52nd Asilomar Conference on Signals, Systems, and Computers.

[49] Win M.Z., Meyer F., Liu Z., Dai W., Bartoletti S., Conti A. (2018). Efficient Multisensor Localization for the Internet of Things: Exploring a New Class of Scalable Localization Algorithms. IEEE Signal Process. Mag..

[50] Chiang K., Tsai G., Chang H., Joly C., EI-Sheimy N. (2019). Seamless navigation and mapping using an INS/GNSS/grid-based SLAM semi-tightly coupled integration scheme. Inf. Fusion.

[51] Leitinger E., Meyer F., Hlawatsch F., Witrisal K., Tufvesson F., Win M.Z. (2019). A Belief Propagation Algorithm for Multipath-Based SLAM. IEEE Trans. Wirel. Commun..

[52] Gentner C., Jost T., Wang W., Zhang S., Dammann A., Fiebig U.C. (2016). Multipath Assisted Positioning with Simultaneous Localization and Mapping. IEEE Trans. Wirel. Commun..

[53] Gentner C., Ulmschneider M., Karásek R., Dammann A. Simultaneous Localization of a Receiver and Mapping of Multipath Generating Geometry in Indoor Environments. Proceedings of the 2021 IEEE Radar Conference (RadarConf21).

[54] Schouten G., Steckel J. RadarSLAM: Biomimetic SLAM using ultra-wideband pulse-echo radar. Proceedings of the 2017 International Conference on Indoor Positioning and Indoor Navigation (IPIN).

[55] Kadambi S., Behboodi A., Soriaga J.B., Welling M., Amiri R., Yerramalli S., Yoo T. (2022). Neural RF SLAM for unsupervised positioning and mapping with channel state information. arXiv.

[56] Leitinger E., Teague B., Zhang W., Liang M., Meyer F. (2022). Data Fusion for Radio Frequency SLAM with Robust Sampling. arXiv.

[57] Ismail K., Liu R., Qin Z., Athukorala A., Lau B.P.L., Shalihan M., Yuen C., Tan U.X. (2022). Efficient WiFi LiDAR SLAM for Autonomous Robots in Large Environments. arXiv.

[58] Arun A., Ayyalasomayajula R., Hunter W., Bharadia D. (2022). P2SLAM: Bearing Based WiFi SLAM for Indoor Robots. IEEE Robot. Autom. Lett..

[59] Gao X., Liu Y., Mu X. SLARM: Simultaneous Localization and Radio Mapping for Communication-aware Connected Robot. Proceedings of the 2021 IEEE International Conference on Communications Workshops (ICC Workshops).

[60] Jirků M., Kubelka V., Reinstein M. WiFi localization in 3D. Proceedings of the 2016 IEEE/RSJ International Conference on Intelligent Robots and Systems (IROS).

[61] Lee Y.C., Yu W. 3D Portable Mapping System to Build Radio Fingerprints and Spatial Map. Proceedings of the 2020 International Conference on Information and Communication Technology Convergence (ICTC).

[62] Yassin A., Nasser Y., Awad M. Geometric Approach in Simultaneous Context Inference, Localization and Mapping using mm-Wave. Proceedings of the 2018 25th International Conference on Telecommunications (ICT).

[63] Iozsa A., Vesa A. The ESPRIT algorithm. Variants and precision. Proceedings of the 2010 9th International Symposium on Electronics and Telecommunications.

[64] Ge Y., Jiang F., Zhu M., Wen F., Svensson L., Wymeersch H. 5G SLAM with Low-complexity Channel Estimation. Proceedings of the 2021 15th European Conference on Antennas and Propagation (EuCAP).

[65] Wen F., Wymeersch H. (2021). 5G Synchronization, Positioning, and Mapping From Diffuse Multipath. IEEE Wirel. Commun. Lett..

[66] Mendrzik R., Meyer F., Bauch G., Win M. Localization, Mapping, and Synchronization in 5G Millimeter Wave Massive MIMO Systems. Proceedings of the 2019 IEEE 20th International Workshop on Signal Processing Advances in Wireless Communications (SPAWC).

[67] Ge Y., Wen F., Kim H., Zhu M., Jiang F., Kim S., Svensson L., Wymeersch H. (2020). 5G SLAM Using the Clustering and Assignment Approach with Diffuse Multipath. Sensors.

[68] Casarrubias-Vargas H., Petrilli-Barceló A., Bayro-Corrochano E. EKF-SLAM and Machine Learning Techniques for Visual Robot Navigation. Proceedings of the 2010 20th International Conference on Pattern Recognition.

[69] Al-Tarras A.E., Yacoub M.I., Asfoor M.S., Sharaf A.M. Experimental Evaluation of Computation Cost of FastSLAM Algorithm for Unmanned Ground Vehicles. Proceedings of the 2019 7th International Conference on Control, Mechatronics and Automation (ICCMA).

[70] Ren J. An improved binocular LSD_SLAM method for object localization. Proceedings of the 2020 IEEE International Conference on Artificial Intelligence and Computer Applications (ICAICA).

[71] Mur-Artal R., Montiel J.M.M., Tardós J.D. (2015). ORB-SLAM: A Versatile and Accurate Monocular SLAM System. IEEE Trans. Robot..

[72] Yuan W., Li Z., Su C.Y. RGB-D sensor-based visual SLAM for localization and navigation of indoor mobile robot. Proceedings of the 2016 International Conference on Advanced Robotics and Mechatronics (ICARM).

[73] Grisetti G., Kümmerle R., Stachniss C., Burgard W. (2010). A Tutorial on Graph-Based SLAM. IEEE Intell. Transp. Syst. Mag..

[74] Andersone I. (2019). Heterogeneous Map Merging: State of the Art. Robotics.

[75] Mirowski P., Ho T.K., Yi S., MacDonald M. SignalSLAM: Simultaneous localization and mapping with mixed WiFi, Bluetooth, LTE and magnetic signals. Proceedings of the International Conference on Indoor Positioning and Indoor Navigation.

[76] Farnham T. Radio environment map techniques and performance in the presence of errors. Proceedings of the 2016 IEEE 27th Annual International Symposium on Personal, Indoor, and Mobile Radio Communications (PIMRC).

[77] Kakalou I., Psannis K., Goudos S.K., Yioultsis T.V., Kantartzis N.V., Ishibashi Y. Radio Environment Maps for 5G Cognitive Radio Network. Proceedings of the 2019 8th International Conference on Modern Circuits and Systems Technologies (MOCAST).

[78] Kaniewski P., Golan E. Localization of Transmitters in VHF Band Based on the Radio Environment Maps Concept. Proceedings of the 2019 Communication and Information Technologies (KIT).

[79] Moore T., Stouch D. A Generalized Extended Kalman Filter Implementation for the Robot Operating System. Proceedings of the 13th International Conference on Intelligent Autonomous Systems (IAS-13).

[80] Särkkä S., Svensson L. Levenberg-Marquardt and Line-Search Extended Kalman Smoothers. Proceedings of the ICASSP 2020—2020 IEEE International Conference on Acoustics, Speech and Signal Processing (ICASSP).

[81] Forster C., Carlone L., Dellaert F., Scaramuzza D. (2017). On-Manifold Preintegration for Real-Time Visual–Inertial Odometry. IEEE Trans. Robot..

[82] Taheribakhsh M., Jafari A., Peiro M.M., Kazemifard N. 5G Implementation: Major Issues and Challenges. Proceedings of the 2020 25th International Computer Conference, Computer Society of Iran (CSICC).

[83] Dellaert F., GTSAM Contributors (2022). GTSAM: Georgia Tech Smoothing and Mapping.

[84] Bao F., Mazokha S., Hallstrom J.O. MobIntel: Passive Outdoor Localization via RSSI and Machine Learning. Proceedings of the 2021 17th International Conference on Wireless and Mobile Computing, Networking and Communications (WiMob).

[85] Ismail M., Mohamad I., Mohd Ali M.A. Availability of GPS and A-GPS signal in UKM campus for hearability check. Proceedings of the 2011 IEEE 10th Malaysia International Conference on Communications.

[86] Borio D., Sokolova N., Lachapelle G. Doppler Measurements and Velocity Estimation: A Theoretical Framework with Software Receiver Implementation. Proceedings of the 22nd International Technical Meeting of the Satellite Division of The Institute of Navigation (ION GNSS).

[87] Karfakis P.T., Couceiro M.S., Portugal D., Cortesão R. UWB Aided Mobile Robot Localization with Neural Networks and the EKF. Proceedings of the 2022 IEEE International Conference on Systems, Man, and Cybernetics (SMC).

[88] Anjum M., Khan M.A., Hassan S.A., Mahmood A., Qureshi H.K., Gidlund M. (2020). RSSI Fingerprinting-Based Localization Using Machine Learning in LoRa Networks. IEEE Internet Things Mag..

